# MSPRP: multi-granularity symptom-aware probabilistic representation prompting for target-domain-free plant disease detection

**DOI:** 10.3389/fpls.2026.1921532

**Published:** 2026-10-29

**Authors:** Xian Cao, Sixiang Quan, Yalu Zhang, Jun Liu, Xianlong Tang, Tai Zhang, Zhihui Wang, Yuankun Wang, Yingying Peng, Huali Li, Xiaoxia Xiao

**Affiliations:** 1 School of Informatics, Hunan University of Chinese Medicine, Changsha, China; 2 AI TCM Lab Hunan, Hunan University of Chinese Medicine, Changsha, China; 3 Second Surveying and Mapping Institute of Hunan Province, Changsha, China; 4 Hunan Engineering Research Center of Natural Ecosystems Carbon Sink Monitoring, Changsha, China; 5 Department of Geography & Geographic Information Science, University of Illinois Urbana-Champaign, Urbana, IL, United States; 6 School of Artificial Intelligence, Hunan University, Changsha, China

**Keywords:** clip, field plant images, plant disease detection, probabilistic prompt learning, symptom-aware representation, target-domain-free learning, vision-language model, zero-shot anomaly detection

## Abstract

**Introduction:**

Existing vision-language zero-shot anomaly detection methods have mainly been validated on industrial datasets and often suffer from lesion-scale variations, domain shifts, and complex background interference when directly applied to field plant images. To address these challenges, this paper proposes MSPRP, a multi-granularity symptom-aware probabilistic representation prompting method for target-domain-free plant disease detection.

**Methods:**

Based on frozen CLIP encoders, MSPRP introduces three modules: the Multi-Granularity Symptom-Aware Module (MGSAM) performs learnable hierarchical fusion and spatial-channel symptom attention on multi-layer patch features, the Uncertainty-Aware Prompt Flow Module (UPFM) models healthy/diseased prompts as probabilistic representations to improve prompt adaptability, and the Adaptive Cross-Modal Prototype Aggregation Module (ACPAM) aggregates suspected symptom prototypes from high-response patches to reduce background interference in image-level disease scoring. The model is trained only on the industrial MVTec AD dataset and tested on target agricultural datasets without target-domain training.

**Results:**

A field Polygonatum odoratum disease dataset is also constructed as an evaluation benchmark. Experimental results show that MSPRP achieves the best average AUROC and AP among methods that do not use target agricultural training images. Ablation results verify the contribution of each module.

**Discussion:**

Overall, MSPRP provides a target-domain-free computational methodology for field plant disease discrimination without target agricultural training images or target-domain pixel-level lesion annotations.

## 1 Introduction

Plant diseases can reduce crop yield and quality (Savary et al., 2019), and for economic crops such as Chinese medicinal herbs, disease symptoms may also affect appearance quality and commercial value.In recent years, convolutional neural networks, Transformer-based models, and related approaches have been applied to disease image recognition (Ferentinos, 2018; Kamilaris and Prenafeta-Boldú, 2018; Mohanty et al., 2016; Saleem et al., 2019; Liu and Wang, 2021), but most methods still require a large number of target-domain training images and their corresponding labels. In real field data collection, illumination, background, imaging distance, and crop growth stage often vary considerably. When new crops, new production areas, or changes in imaging devices are encountered, it is often difficult to collect complete target-domain training data in time. Therefore, methods that can directly perform disease discrimination without relying on target-domain training data are more suitable for such field application scenarios.

Anomaly detection provides a feasible solution for low-annotation disease anomaly discrimination. Methods such as PaDiM, PatchCore, and SimpleNet are commonly used in industrial anomaly detection (Defard et al., 2021; Roth et al., 2022; Liu et al., 2023), and many of them model normal feature distributions using target-domain normal training samples. In other words, although these methods do not necessarily require a large number of abnormal samples, they still need target-domain normal samples for modeling. This requirement still introduces additional data collection pressure. Therefore, relying solely on traditional anomaly detection methods cannot fully address the lack of target-domain training data.

Vision-language models provide a new direction for target-domain-free anomaly detection. CLIP maps image and text features into a shared semantic space (Radford et al., 2021), enabling normal/abnormal semantic matching through textual descriptions. Based on this capability, methods such as WinCLIP (Jeong et al., 2023), AnomalyCLIP (Zhou et al., 2024), and TPS (Ma et al., 2025)have explored CLIP-based zero-shot anomaly detection. These methods reduce the dependence on target-domain training data, but most of them have still been validated mainly on industrial images. Field images differ substantially from industrial images, as they often contain illumination changes, viewpoint variations, weeds, soil, occlusion, and other forms of interference, while lesion regions may occupy only local areas of leaves. In such scenarios, fixed textual prompts may suffer from insufficient adaptability, and disease responses from small lesion regions may also be weakened by background patches. Therefore, zero-shot disease detection in complex field scenarios cannot rely solely on general image-text alignment, but also needs to address lesion scale variations, insufficient prompt adaptation, and complex background interference.

To address these issues, MSPRP is proposed as a multi-granularity symptom-aware probabilistic representation prompting method for target-domain-free plant disease detection. The proposed method introduces three modules based on frozen vision-language encoders. The Multi-Granularity Symptom-Aware Module is designed to address lesion-scale variations, the Uncertainty-Aware Prompt Flow Module alleviates the limited adaptability of fixed prompts, and the Adaptive Cross-Modal Prototype Aggregation Module reduces the interference of complex backgrounds on image-level disease scores. During training, only the MVTec AD source-domain data are used, while target plant disease datasets are not involved in training.

Accordingly, the main contributions of this paper are summarized as follows:

1. MSPRP is constructed as a multi-granularity symptom-aware probabilistic representation prompting method for target-domain-free plant disease detection, enabling image-level healthy/diseased discrimination in complex field scenarios.
2. A Multi-Granularity Symptom-Aware Module (MGSAM) is designed to enhance symptom-related visual cues at different scales through learnable fusion of multi-layer patch features and spatial-channel symptom attention.
3. An Uncertainty-Aware Prompt Flow Module (UPFM) is developed to model healthy/diseased prompts as probabilistic representations, thereby alleviating the insufficient adaptability of fixed prompts to field images.
4. An Adaptive Cross-Modal Prototype Aggregation Module (ACPAM) is designed to aggregate high-response visual prototypes from local patches and enhance cross-modal feature interaction, thereby reducing the dilution of locally discriminative cues by numerous background patches in image-level disease scoring.
5. A real-field *Polygonatum odoratum* disease evaluation dataset is organized and combined with three additional target agricultural datasets to evaluate the zero-shot transfer ability and cross-domain generalization performance of the model from the industrial source domain to field agricultural target domains.

## 2 Related work

### 2.1 Anomaly detection in field scenarios

Agricultural anomaly detection is an important research direction in precision agriculture. Gkountakos et al (Gkountakos et al., 2024)systematically reviewed deep learning-based anomaly detection methods in precision agriculture and categorized them from the perspectives of agricultural applications, anomaly types, model architectures, and supervision strategies, indicating that agricultural anomalies include not only plant diseases but also environmental stress, pests, weeds, and yield abnormalities, with substantial differences in task formulation, data modality, and supervision setting. Wei et al (Wei et al., 2025)proposed an anomaly detection framework for precision field crop protection, integrating UAV, satellite, ground sensor, and meteorological data for multimodal anomaly monitoring. These studies indicate that agricultural anomaly detection has evolved from single visual feature modeling to deep learning and multimodal spatiotemporal modeling; however, many methods still rely on target-scene data, multi-sensor inputs, or specific agricultural datasets for training and adaptation, making them difficult to directly satisfy the requirements of target-domain-free image-level anomaly detection.

In image anomaly detection, existing studies have attempted to transfer unsupervised or label-free methods from industrial anomaly detection to agricultural images. Yang et al (Yang et al., 2026)adapted EfficientAD to small agricultural datasets and validated the effectiveness of a label-free anomaly detection framework on PlantVillage, Coffee Leaf, and real sweet potato datasets. This study suggests that industrial anomaly detection frameworks can provide a methodological basis for agricultural image anomaly detection, although complex backgrounds, reflections, and irregular lesion boundaries in agricultural images may still affect anomaly localization and model stability. Defard et al (Defard et al., 2021). proposed PaDiM, which performs anomaly detection and localization by modeling patch-level feature distributions; Roth et al (Roth et al., 2022)proposed PatchCore, which improves anomaly detection performance through a local feature memory bank; and Liu et al (Liu et al., 2023)proposed SimpleNet, which performs anomaly detection using feature perturbation and a discriminative network. These methods have achieved favorable results in industrial anomaly detection, but they typically require target-domain normal samples to build normal feature repositories or estimate normal distributions. When applied to complex agricultural field images, the acquisition, screening, and modeling of target-domain normal samples still introduce additional costs, making these methods difficult to directly meet target-domain-free application requirements.

Compared with traditional anomaly detection methods that rely on target-domain normal samples, CLIP-based zero-shot anomaly detection provides a new direction for target-domain-free agricultural anomaly detection. Radford et al (Radford et al., 2021). proposed CLIP, which maps images and texts into a unified semantic space through large-scale image-text contrastive learning. Jeong et al (Jeong et al., 2023). proposed WinCLIP, which combines state-word prompts, template ensembling, and local window matching for zero-shot and few-shot anomaly detection. Zhou et al (Zhou et al., 2024). proposed AnomalyCLIP, which constructs general normal and abnormal prompts through object-agnostic prompt learning to reduce the dependence of the model on specific category semantics. Chen et al (Chen et al., 2025). proposed CLIP-AD, which addresses zero-shot anomaly detection using a language-guided dual-path architecture. Ma et al (Ma et al., 2025). proposed TPS, which establishes cross-modal alignment at different granularities through global prompts and shunt prompts. Overall, CLIP-based methods reduce the dependence on target-domain training data and are more aligned with application scenarios in complex fields where sufficient target crop samples are difficult to collect. However, most of these methods have still been validated mainly on industrial anomaly detection datasets. When directly transferred to agricultural target domains, further efforts are still needed to address cross-domain prompt adaptation, agricultural symptom representation, and insufficient local anomaly responses.

### 2.2 Prompt learning and uncertainty modeling

In CLIP-based methods, textual prompts directly affect the matching results between image features and text features. Hand-crafted prompts usually need to be designed in advance, and model performance can be sensitive to word selection and template formulation. Zhou et al (Zhou et al., 2022). proposed CoOp, which replaces manually designed prompts with learnable context vectors to better adapt vision-language models to downstream tasks. Zhou et al (Zhou et al., 2024). further introduced learnable prompts in AnomalyCLIP to represent normal and abnormal semantics, thereby improving the semantic modeling of anomalous states. These approaches reduce the dependence on manually designed prompts, but their learned prompt representations are deterministic after training. For field plant images with substantial variations in illumination, background, imaging distance, and symptom appearance, a single deterministic prompt representation may be insufficient to describe the variability of healthy and diseased visual patterns.Uncertainty modeling provides an alternative perspective for prompt representation. Kingma and Welling (Kingma and Welling, 2014) proposed VAE, which learns latent variable distributions through the reparameterization trick and provides a foundation for probabilistic representation learning. Qu et al (Qu et al., 2025). proposed Bayes-PFL, which introduces Bayesian modeling into zero-shot anomaly detection and formulates the prompt space as a learnable probability distribution. These studies indicate that prompt representations can be extended from deterministic vectors to distributional representations that preserve a certain degree of variability.

Based on this probabilistic representation perspective, UPFM models healthy and diseased prompt contexts as learnable probability distributions rather than single deterministic context vectors. During source-domain training, reparameterized sampling is used to introduce representation variability, while the distribution means are used during inference to obtain deterministic prompt representations. Unlike conventional deterministic prompt learning, UPFM explicitly represents uncertainty in healthy/diseased prompt contexts. Meanwhile, no target agricultural images are used for prompt adaptation or parameter updating, so the probabilistic prompt modeling remains consistent with the target-domain-free setting of this study.

### 2.3 Symptom-aware multi-granularity visual representation

Anomalous information in field disease images usually appears as local symptoms rather than complete and clearly defined objects. Lesions may appear as small spots, yellowing leaf margins, local necrosis, or changes in leaf surface texture, and different symptoms may exhibit substantial scale variations in images. Lin et al (Lin et al., 2017). proposed FPN, which enhances multi-scale visual representation by fusing features from different levels. Woo et al (Woo et al., 2018). proposed CBAM, which computes attention along both channel and spatial dimensions to refine feature responses. Peng et al (Peng et al., 2025). proposed MGANet, which introduces multi-granularity information into remote sensing few-shot segmentation to address visual differences among samples. These studies indicate that multi-level features and attention mechanisms can complement visual information at different scales. The multi-granularity symptom-aware module in this paper mainly operates on the visual branch, performing learnable hierarchical fusion on multi-layer patch features from the frozen visual encoder and enhancing symptom-related visual cues through spatial-channel symptom attention.

### 2.4 Cross-modal visual prototype aggregation

Lesion regions in field images usually occupy only local areas, whereas background patches such as healthy leaves, soil, weeds, and shadows are much more numerous. If the model directly relies on global image-text matching, local symptom information may be weakened by background information. Venkatraman et al (Venkatraman et al., 2026). proposed SPROUT, which constructs prototypes around symptom regions in few-shot precision agriculture tasks through symptom-centric prototypical representation optimization and uncertainty-aware tuning. This study indicates that prototype representations of symptom-related regions can provide effective cues for low-annotation agricultural image recognition. The adaptive cross-modal prototype aggregation module in this paper further operates on the vision-text fusion branch, aggregating potential symptom prototypes from high-response patches and feeding prototype information back into text features and patch features to reduce the interference of background patches on image-level disease scores. This module is not designed for pixel-level lesion segmentation, but for image-level healthy/diseased discrimination.

## 3 Method

### 3.1 Problem definition and overall framework

This paper focuses on a specific problem in real field scenarios: target-domain-free plant disease detection in agricultural field scenarios. Given an agricultural image *x* as input, the model is required to determine whether *x* contains disease-related signals. This study focuses on image-level disease discrimination, without identifying specific disease categories or performing complete lesion segmentation. The source-domain dataset is denoted as $D_{s}={\left\{\left(x_{i}^{s},\;y_{i}^{s},\;m_{i}^{s}\right)\right\}}_{i=1}^{N_{s}}$, where $x_{i}^{s}$ denotes a source-domain image, $y_{i}^{s}\in \left\{0,\;1\right\}$ denotes the image-level normal or anomalous label, and $m_{i}^{s}\in {\left\{0,\;1\right\}}^{H\times W}$ denotes the source-domain anomaly mask. The mask of a normal sample is an all-zero map. The target agricultural test set is denoted as $D_{t}={\left\{\left(x_{j}^{t},\;y_{j}^{t}\right)\right\}}_{j=1}^{N_{t}}$, where $x_{j}^{t}$ denotes a target agricultural image and $y_{j}^{t}$ denotes its image-level label.

The overall architecture of MSPRP is illustrated in **Figure 1** and mainly consists of a visual branch, a textual branch, and a cross-modal aggregation branch. The visual branch adopts a frozen CLIP visual encoder to extract multi-layer patch features, and then applies the Multi-Granularity Symptom-Aware Module (MGSAM) for learnable hierarchical fusion and spatial-channel symptom attention enhancement, thereby supplementing local disease cues at different scales. The textual branch constructs two groups of healthy/diseased textual prompts at the object and part levels based on a frozen CLIP text encoder, and models the prompt contexts as probabilistic representations through the Uncertainty-Aware Prompt Flow Module (UPFM) to alleviate the insufficient adaptability of fixed prompts to field images. The cross-modal aggregation branch further introduces the Adaptive Cross-Modal Prototype Aggregation Module (ACPAM), which aggregates suspected symptom prototypes from high-response patches and feeds them back into textual features and patch features, thereby reducing the interference of background patches, such as healthy leaves, soil, weeds, and shadows, on image-level disease scores. Finally, the model calculates local disease responses based on the enhanced visual and textual features, obtains the image-level disease score through Top-*K* multi-instance scoring, and outputs the healthy/diseased discrimination result. During training, the model is optimized only on the MVTec AD source-domain data, while target agricultural images are used only for zero-shot inference and evaluation and are not involved in any parameter updates. In **Figure 1**, the black solid lines denote the forward paths shared by the training and inference stages, while the red dashed lines indicate the paths involved in loss computation and learnable module parameter updates during source-domain training.

**Figure 1:**
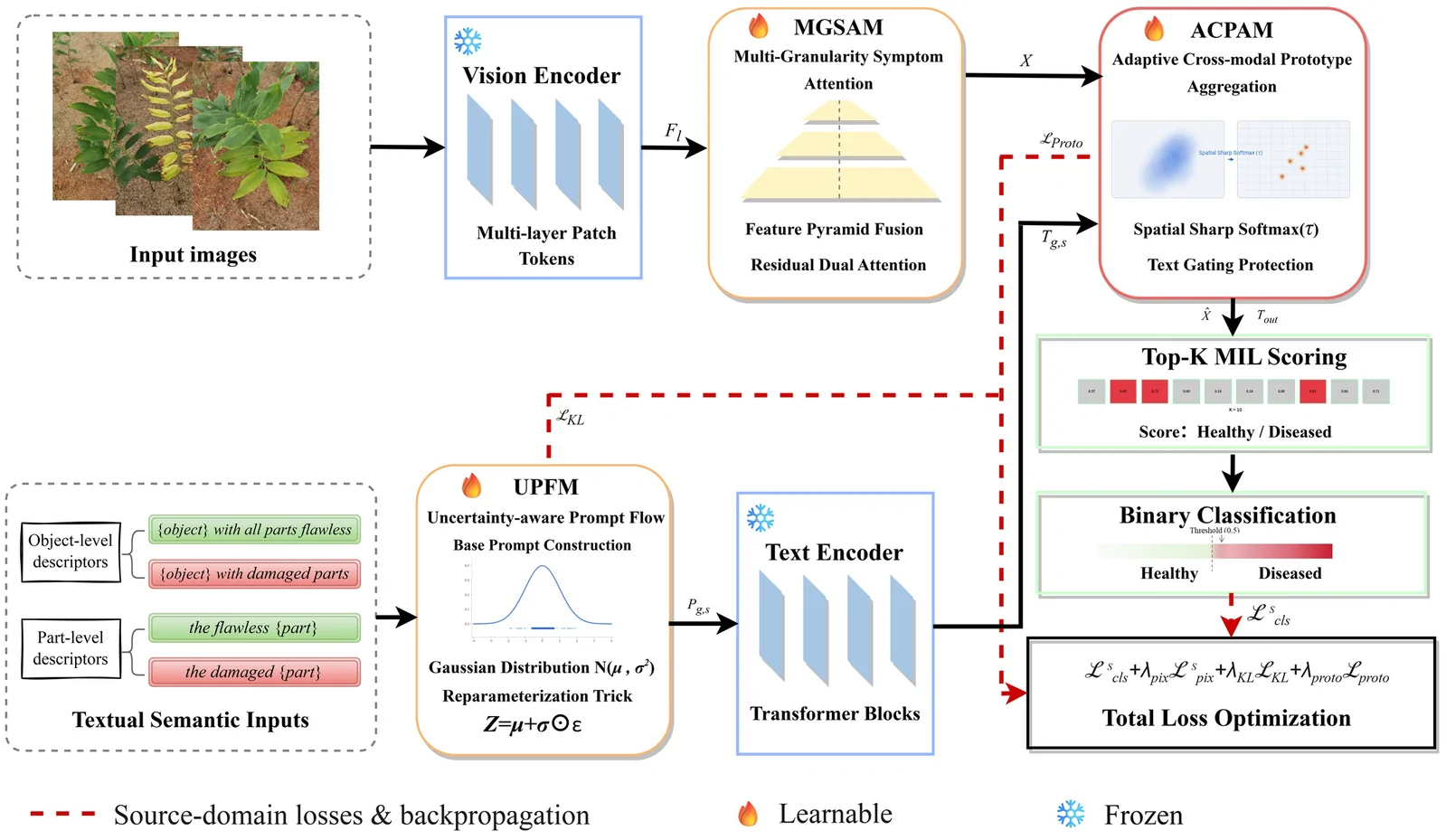
Overall framework of MSPRP.

### 3.2 Multi-granularity symptom-aware module

Field disease symptoms exhibit substantial scale variations across different samples and disease stages, and may appear as small spots, local texture changes, leaf margin chlorosis, local necrosis, or large-area lesions. If only single-level patch features are used, the model may miss small-scale symptoms or provide insufficient representation for large abnormal regions. To address the insufficient representation of local symptoms caused by lesion scale variations, a Multi-Granularity Symptom-Aware Module is designed, as shown in **Figure 2**. This module integrates visual information from multiple hierarchical levels and enhances symptom-related spatial regions and feature channels, thereby providing more discriminative visual representations for subsequent image-level disease discrimination.

**Figure 2:**
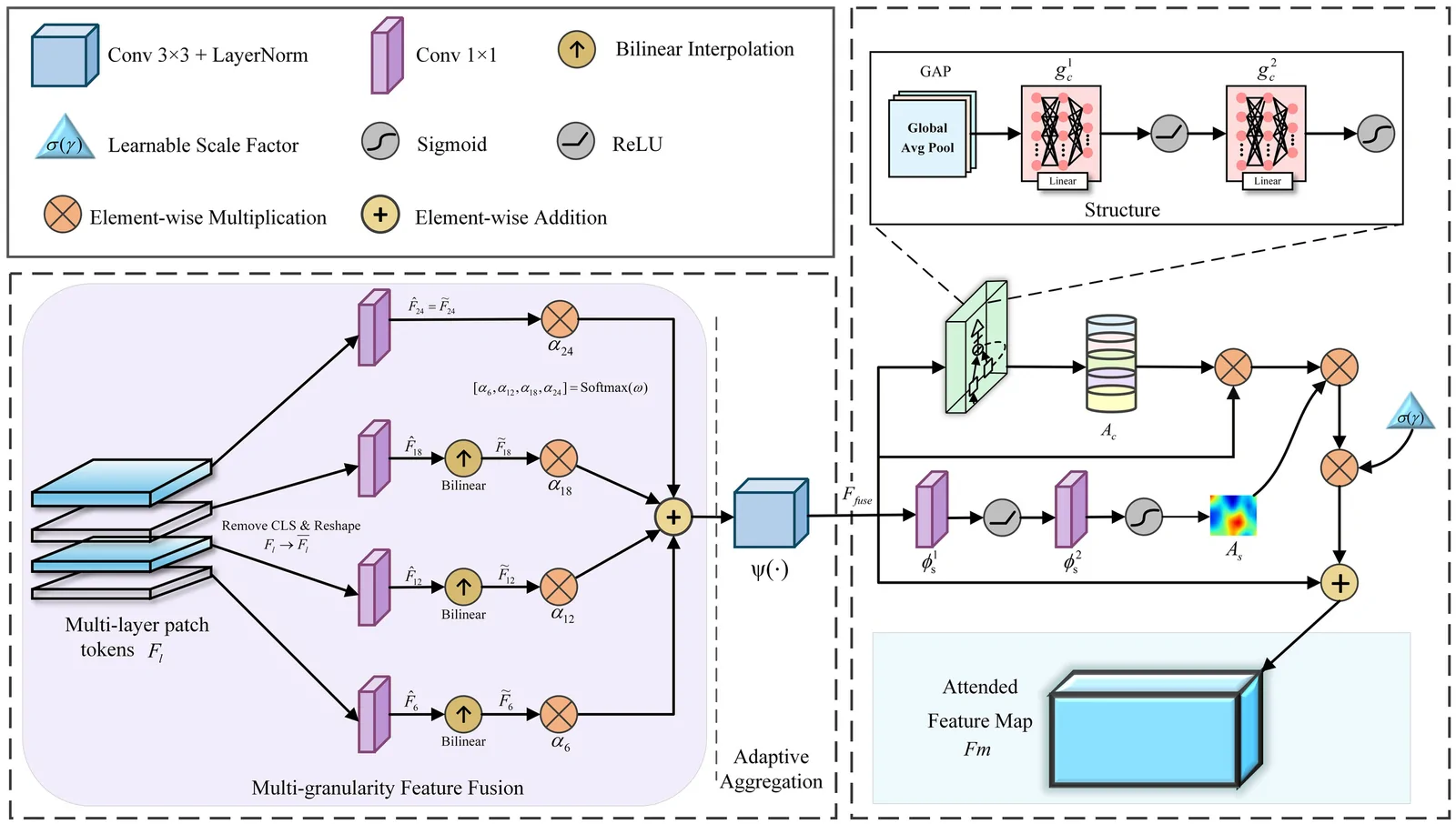
Structure of the multi-granularity symptom-aware module.

Given the multi-layer patch tokens output by the frozen visual encoder, this paper selects the features from the 6th, 12th, 18th, and 24th layers for multi-granularity modeling, and denotes the selected layer set as S={6, 12, 18, 24}. These layers are approximately evenly distributed across the depth of the ViT-L/14 visual encoder and therefore provide representations from different semantic depths. Relatively shallow layers retain more local appearance information, such as edges, textures, and fine structural variations, whereas deeper layers encode increasingly abstract and globally contextualized visual semantics. Sampling the 6th, 12th, 18th, and 24th layers provides a simple way to cover shallow, intermediate, and deep representation levels without introducing dense multi-layer extraction and excessive computational redundancy. The selected features are subsequently adaptively fused in MGSAM to combine complementary local-detail and high-level semantic information for symptom representation. For any selected layer l∈S, its output feature is denoted as $F_{l}\in {\mathbb{R}}^{B\times (N+1)\times C}$, where *B* is the batch size, *N* is the number of patches, and *C* is the channel dimension. Each layer feature contains one class token and *N* patch tokens. After removing the class token from each layer, the patch tokens are first reshaped into a two-dimensional feature map, as shown in Equation 1:

(1) $$\bar{F_{l}}=\text{Reshape}\left(F_{l}\left[:,\;1:,\;:\right]\right)$$

To fuse features from different levels, a 1×1 convolution is used to project the features of each two-dimensional feature map $\bar{F_{l}}$ into a unified channel dimension:

(2) $$\hat{F_{l}}=\phi_{l}(\bar{F_{l}})$$

In Equation 2, $\phi_{\text{l}}\left(\cdot \right)\;$ denotes a convolution operation used to align the channel dimensions. Meanwhile, to ensure that features from different layers have consistent spatial sizes during fusion, this paper uses the spatial resolution corresponding to the 24th-layer patch feature as the target resolution, denoted as $Size_{tar}=Size\left({\hat{F}}_{24}\right)$. Subsequently, the remaining selected-layer features are aligned to this target spatial size through bilinear interpolation, as shown in Equation 3:

(3) $$\tilde{F_{l}}=\text{Up}\left(\hat{F_{l}}\right)$$

Since features from different levels contain different symptom-related information, learnable hierarchical weights are adopted to achieve adaptive fusion. The implementation is formulated in Equations 4, 5 as follows:

(4) $$\alpha_{l}\;=\frac{\exp(\omega_{l})}{\sum_{r\in S}\exp(\omega_{r})},\;l\in S$$

(5) $$F_{fuse}=\psi (\underset{l\in S}{\sum }\alpha_{l}\tilde{F_{l}})$$

Here, *ω_l_* denotes the learnable parameter of the *l*-th layer, *α_l_* denotes the corresponding hierarchical weight, and $\underset{l\in S}{\sum }\alpha_{l}=1$. ψ(·) denotes a 3×3 convolution followed by layer normalization, which is used to further aggregate the weighted fused features, thereby integrating symptom information from different levels and enhancing the spatial consistency of the fused features. The fused features are then fed into a residual spatial-channel symptom attention module, which consists of a spatial attention branch, a channel attention branch, and a residual enhancement path. The computation is formulated in Equations 6–8 as follows:

(6) $$A_{s}=\sigma \left(\phi_{s}^{2}\left(\delta \left(\phi_{s}^{1}\left(F_{fuse}\right)\right)\right)\right)$$

(7) $$A_{c}=\sigma \left(g_{c}^{2}\left(\delta \left(g_{c}^{1}\left(\text{GAP}\left(F_{fuse}\right)\right)\right)\right)\right)$$

(8) $$F_{m}=F_{fuse}+\sigma \left(\gamma \right)\left(A_{s}⊙A_{c}⊙F_{fuse}\right)$$

Here, *A_s_* denotes the spatial attention map, *A_c_* denotes the channel attention vector, $\phi_{\text{s}}^{1}$ and $\phi_{s}^{2}$ denote the 1×1 convolutions in the spatial attention branch, and $g_{c}^{1}$ and $g_{c}^{2}$ denote the linear transformations in the channel attention branch. δ(·) denotes the ReLU function, GAP(·) denotes global average pooling, σ(·) denotes the Sigmoid function, and ⊙ denotes element-wise multiplication. During computation, *A_s_* is broadcast along the channel dimension, while *A_c_* is broadcast along the spatial dimensions. γ is a learnable parameter, and σ(γ) denotes the residual scaling weight constrained by the Sigmoid function, which is used to control the strength of the attention enhancement term. Through this residual spatial-channel symptom attention unit, the model can enhance symptom-related regions and channel responses while preserving the original fused features, reducing excessive suppression of the original features caused by attention gating. The resulting *F_m_* is then flattened into patch tokens and fed into the subsequent cross-modal prototype aggregation module.

### 3.3 Uncertainty-aware prompt flow module

Fixed-form textual prompts suffer from insufficient adaptability in the target agricultural domain. Field images are affected by factors such as illumination, background, shooting distance, and growth status, causing samples from the same healthy or suspected diseased category to exhibit variations in visual appearance. If prompt representations always remain in a single deterministic form, the model can be affected by the limited expressive range of prompts during cross-domain testing. To alleviate this problem, an Uncertainty-Aware Prompt Flow Module is designed, as illustrated in **Figure 3**.This module extends the learnable contexts in prompts from deterministic vectors to probabilistic representations, allowing healthy and diseased semantics to retain a certain range of variation during training, thereby improving the adaptability of textual prompts to complex field images.

**Figure 3:**
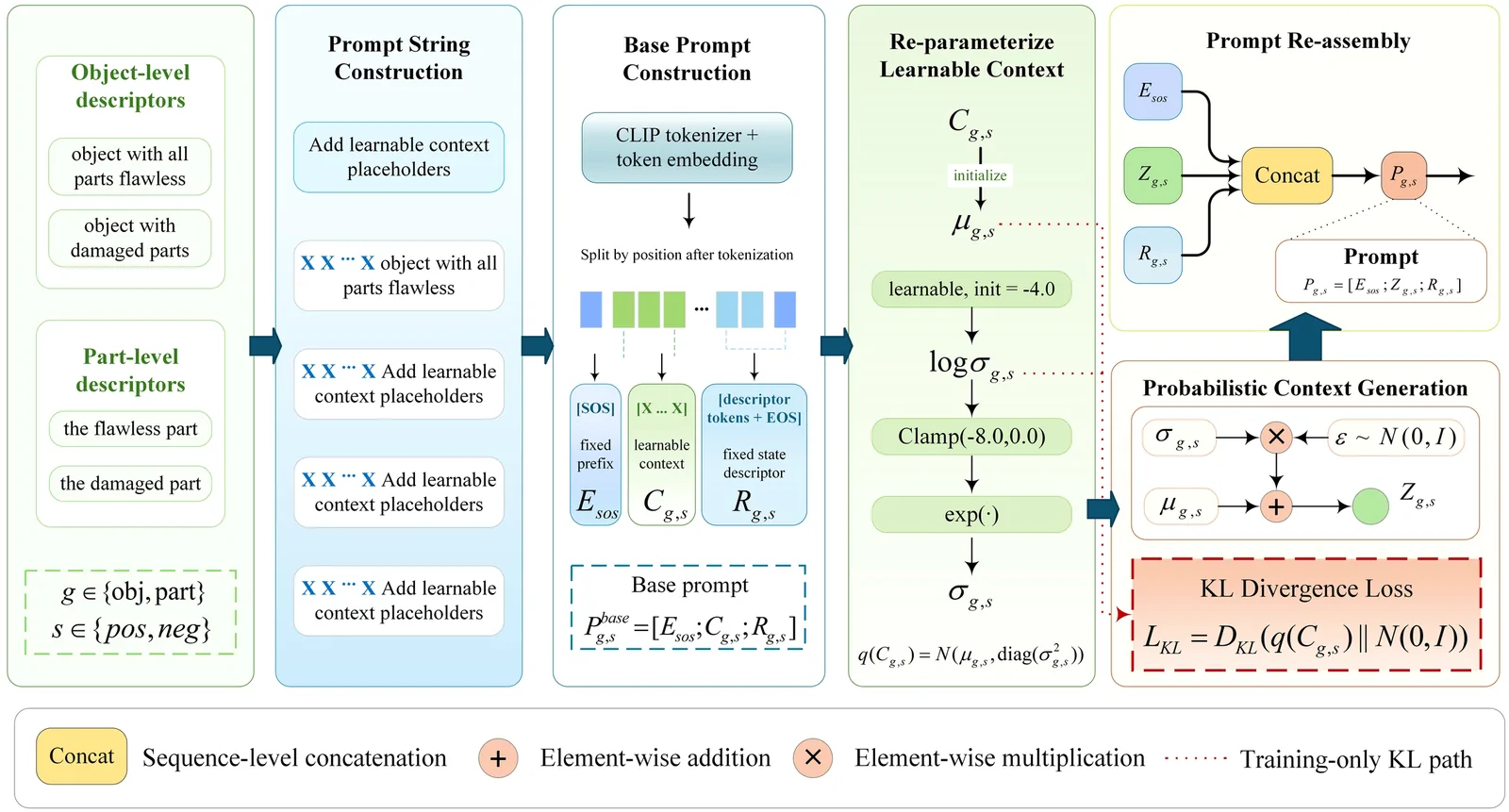
Structure of the uncertainty-aware prompt flow module.

The input of UPFM is a set of textual semantic descriptions. This paper defines two groups of textual descriptions, corresponding to object-level semantics and local part-level semantics, respectively. The object-level descriptions are used to represent the healthy or diseased state of the entire target, whereas the part-level descriptions describe the healthy or diseased state of local regions. The prompt granularity is denoted as g∈{obj, part}, where obj denotes the object-level prompt and part denotes the part-level prompt. The state category is denoted as s∈{pos, neg}, where pos represents healthy semantics and neg represents disease semantics. The corresponding fixed state description is denoted as *R_g, s_*. For example, *R*_obj, pos_ denotes “object with all parts flawless”, while *R*_part, pos_ denotes “the flawless part”.

In the base prompt construction stage, a set of learnable context placeholders *X* is first inserted before the fixed state description to form a prompt string with context placeholders. The prompt string is then processed by the CLIP tokenizer and token embedding, and is split according to token positions into three parts: the fixed prefix, the learnable context positions, and the fixed state description. The base prompt can be formulated as $P_{g,\;s}^{base}=\left[E_{sos};C_{g,\;s};R_{g,\;s}\right]$, where *E_sos_* denotes the fixed start token in the CLIP text sequence, *C_g, s_* denotes the learnable context tokens corresponding to the placeholder *X*, and *R_g, s_* denotes the fixed state description tokens. It should be noted that *X* itself does not carry specific semantics; it only serves to reserve the positions of learnable contexts, while the corresponding context vector *C_g, s_* is the actual component involved in training. Next, *C_g, s_* is used to initialize the mean *μ_g, s_*, and a learnable parameter log σ*_g, s_* is introduced with an initial value of -4.0. To ensure numerical stability, log σ*_g, s_* is clipped and then transformed by an exponential operation to obtain the standard deviation. Accordingly, the standard deviation and probability distribution of the learnable context are formulated in Equations 9 and 10 as follows:

(9) $$\sigma_{g,\;s}=\exp\left(\text{Clamp}\left(\log\;\sigma_{g,\;s},\;-8.0,\;0.0\right)\right)$$

(10) $$q\left(C_{g,\;s}\right)=N\left(\mu_{g,\;s},\;\text{diag}\left(\sigma_{g,\;s}^{2}\right)\right)$$

Here, Clamp(·) denotes a clipping function used to constrain log σ*_g, s_* within the range of [−8.0, 0.0]. Values smaller than −8 are clipped to −8, whereas values larger than 0 are clipped to 0. This prevents the prompt distribution from degenerating due to an excessively small standard deviation and avoids training instability caused by an excessively large standard deviation.

Training process During training, UPFM generates stochastic context representations from the context distribution using the reparameterization trick. Specifically, noise *ϵ_g, s_* is first sampled from a standard normal distribution and then combined with the context distribution parameters *μ_g, s_* and σ*_g, s_* to obtain the probabilistic context *z_g, s_* , as formulated in Equations 11 and 12:

(11) $$\epsilon_{g,\;s}\sim N\left(0,\;I\right)$$

(12) $$z_{g,\;s}=\mu_{g,\;s}+\sigma_{g,\;s}⊙\epsilon_{g,\;s}$$

Here, *ϵ_g, s_* is generated by a random function in implementation, with each element in the tensor independently sampled from the standard normal distribution, and ⊙ denotes element-wise multiplication. Through the reparameterization formulation, the random sampling process can be expressed as a differentiable function of *μ_g, s_* and σ*_g, s_*, allowing the sampled context *z_g, s_* to participate in backpropagation. Subsequently, the obtained probabilistic prompt context *z_g, s_* is concatenated with the fixed prefix and fixed suffix along the sequence dimension to form complete healthy and diseased prompts, which are then fed into the frozen text encoder to obtain textual features, as shown in Equations 13 and 14:

(13) $$P_{g,\;s}=\text{Concat}(E_{sos},\;z_{g,\;s},\;R_{g,\;s})$$

(14) $$T_{g,\;s}=E_{txt}(P_{g,\;s})$$

Here, Concat(·) denotes the concatenation operation along the sequence dimension, and $E_{txt}\left(\cdot \right)$ denotes the frozen CLIP text encoder. After encoding, object-level healthy textual features, object-level diseased textual features, part-level healthy textual features, and part-level diseased textual features are obtained for subsequent matching between image patch features and healthy/diseased semantics.

To prevent the context distribution from deviating excessively, a KL divergence constraint is imposed on the learnable context distribution during training.This constraint is applied to the context distribution determined by *μ_g, s_* and σ*_g, s_*, and is formulated in Equation 15 as follows:

(15) $$L_{KL}=\sum_{g\in \{obj,\;part\}}\sum_{s\in \{pos,\;neg\}}D_{KL}(q(C_{g,\;s})∥N(0,\;I))$$

The detailed formulation of *q*(*C_g, s_*) is given in Equation 10.This constraint limits the variation range of the healthy and diseased prompt distributions, allowing the prompt contexts to retain a certain degree of stochasticity while preventing them from excessively deviating from a stable semantic space.

Inference process During inference, stochastic sampling is disabled and the learned distribution mean *μ_g, s_* is directly used as the prompt context representation. Therefore, the formal inference procedure is deterministic and does not require Monte Carlo sampling. The learned standard deviation σ*_g, s_* affects model optimization during source-domain training by controlling the stochastic variation of the sampled prompt contexts, whereas only the learned distribution mean is used to construct the final healthy and diseased prompts during evaluation.This design separates stochastic representation learning from deterministic prediction, avoiding run-to-run fluctuations caused by random prompt sampling at test time.

Overall, UPFM does not generate specific disease category names; instead, it models the healthy and diseased prompt contexts as probability distributions, allowing the textual prompts to maintain a certain range of variation and thereby alleviating the insufficient adaptability of fixed prompts to the target agricultural domain.

### 3.4 Adaptive cross-modal prototype aggregation module

In complex field images, suspected diseased regions usually occupy only a small portion of the image, whereas background patches such as healthy leaves, soil, weeds, and shadows are much more numerous. If all patches are directly used for image-level discrimination, local disease responses can be easily weakened by large amounts of background information. To address the dilution of local disease cues by background patches, an Adaptive Cross-Modal Prototype Aggregation Module is designed, as shown in **Figure 4**. This module takes the patch features output by MGSAM as input, first aggregates a small set of high-response visual prototypes from the patch features, and then uses these prototypes to enhance both text features and patch features, thereby emphasizing locally discriminative visual information for subsequent image-level disease discrimination.In the following cross-modal aggregation stage, the object-level textual features are referred to as global textual features *T_g_*, whereas the part-level textual features are referred to as shunt textual features *T_s_*.

**Figure 4:**
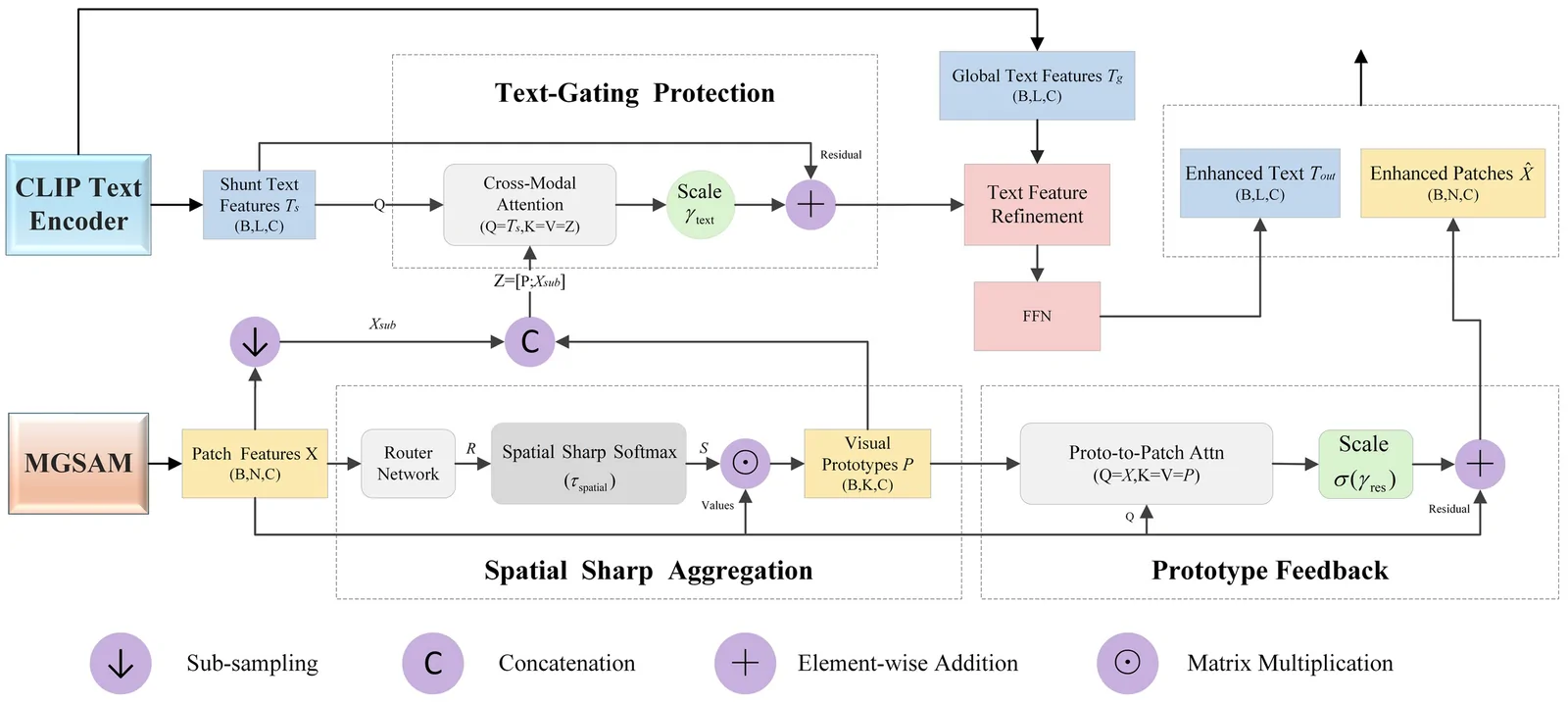
Structure of the adaptive cross-modal prototype aggregation module.

Let the patch features output by MGSAM be denoted as $X={\left\{x_{n}\right\}}_{n=1}^{N}$, where $X\in {\mathbb{R}}^{B\times N\times C}$, *B* denotes the batch size, *N* denotes the number of patches, *C* denotes the channel dimension, and $x_{n}\in {\mathbb{R}}^{C}$ represents the feature vector of the *n*-th patch. For simplicity of notation, the batch dimension is omitted in the following equations except when tensor dimensions are specified. To adaptively aggregate potential symptom regions from a large number of patches, a routing network is first used to predict the assignment scores between each patch and different visual prototypes, followed by sharpened aggregation along the spatial dimension, as formulated in Equations 16–19:

(16) $$R=\text{Router}\left(X\right),\;R\in {\mathbb{R}}^{N\times K}$$

(17) $$S_{n,\;k}=\frac{\exp(R_{n,\;k}/\tau_{spatial})}{\sum_{r=1}^{N}\exp(R_{r,\;k}/\tau_{spatial})}$$

(18) $$P_{k}=\overset{N}{\underset{n=1}{\sum }}S_{n,\;k}\;x_{n}$$

(19) $$P=[P_{1},\;P_{2},\;\ldots ,\;P_{K}],\;P\in {\mathbb{R}}^{K\times C}$$

Here, Router(·)  denotes a lightweight network used to generate prototype assignment scores, *K* denotes the number of visual prototypes, and *R_n, k_* denotes the score of assigning the *n*-th patch to the *k*-th visual prototype. *S_n,k_* denotes the spatial aggregation weight of the *n*-th patch for the *k*-th visual prototype, and *τ*_spatial_ is the spatial temperature coefficient. A smaller *τ*_spatial_ makes the weight distribution more concentrated, causing the visual prototypes to be formed more preferentially from a small number of high-response patches. *P_k_* denotes the *k*-th visual prototype.It should be noted that the prototype router operates only on the visual patch features *X* and is not directly supervised by disease-category annotations or healthy/diseased textual semantics. Therefore, the resulting prototypes should be interpreted as latent high-response visual representations rather than prototypes with explicitly assigned symptom meanings. The spatially sharpened aggregation encourages each prototype to concentrate on a subset of visually responsive patches instead of uniformly averaging all image patches. In diseased images, these patches may correspond to lesion-related textures or structural changes; however, visually salient background structures, such as soil regions, shadows, leaf boundaries, or strong illumination changes, may also receive high routing responses. Healthy/diseased semantic information is introduced subsequently through the cross-modal interaction between the visual prototypes and textual features. Thus, ACPAM is designed to increase the contribution of locally discriminative visual regions, but it does not guarantee that every learned prototype corresponds exclusively to a disease symptom.

After obtaining the visual prototypes P, these prototypes are further used to perform cross-modal enhancement of the text features and patch features. According to the global-shunt prompt setting, the text branch obtains two groups of text features after UPFM: the shunt text feature *T_s_* and the global text feature *T_g_*. Specifically, $T_{s\;}=\left[T_{pos}^{s};T_{neg}^{s}\right]$ is encoded from the healthy/diseased shunt prompts and is used for cross-modal alignment with local patch features. $T_{g\;}=\left[T_{pos}^{g};T_{neg}^{g}\right]$ is encoded from the healthy/diseased global prompts and is used to preserve image-level healthy and diseased semantics. Here, *T_s_*, $T_{g}\in {\mathbb{R}}^{L_{t}\times C}$, where *L_t_* denotes the number of textual prompt features corresponding to the two textual semantics of health and disease, and *C* denotes the feature dimension. To preserve part of the original patch information, $X_{sub}\in {\mathbb{R}}^{M\times C}$ is sampled from *X*, where *M* denotes the number of sampled patches. It is then concatenated with the visual prototypes along the token dimension, as shown in Equations 20–24:

(20) $$Z=[P;X_{sub}],\;Z\in {\mathbb{R}}^{(K+M)\times C}$$

(21) $${\hat{T}}_{s}=T_{s}+\sigma \left(\gamma_{text}\right)\cdot \text{Attn}\left(Q=T_{s},\;K=Z,\;V=Z\right)$$

(22) $$T_{r}=\text{TextRefine}\left({\hat{T}}_{s},\;T_{g}\right)={\hat{T}}_{s}+\sigma \left(\gamma_{\text{cross}}\right)\cdot \text{Attn}\left(Q={\hat{T}}_{s},\;K=T_{g},\;V={\hat{T}}_{s}\right)$$

(23) $$T_{out}=T_{r}+FFN(\text{LN}(T_{r}))$$

(24) $$\hat{X}=X+\sigma \left(\gamma_{res}\right)\cdot \text{Attn}\left(Q=X,\;K=P,\;V=P\right)$$

Here, []; denotes concatenation along the token dimension, and Z denotes the cross-modal enhancement sequence composed of visual prototypes and sampled patches. In Equation 21, Attn(·) denotes the multi-head attention operation, where *T_s_* serves as the query and *Z* serves as both the key and value, enabling visual information from suspected symptom regions to be injected into the shunt textual features. ${\hat{T}}_{s}$ denotes the enhanced shunt text feature, γ_text_ is a learnable gating parameter in the text enhancement branch, and σ(γ_text_) denotes the text residual scaling weight constrained by the Sigmoid function, which controls the strength of visual information injected into the text features.

In Equation 22, γ_cross_ is a learnable gating parameter, and σ(γ_cross_) controls the strength of the global-text-guided cross-attention update applied to the enhanced shunt textual features. TextRefine(·)  denotes the textual feature updating operation; specifically, the model performs attention computation by using the enhanced shunt textual features ${\hat{T}}_{s}$ as the query and value, and the global textual features *T_g_* as the key.This process does not directly replace the shunt textual features with the global textual features, but instead uses *T_g_* to provide global healthy/diseased semantic matching cues, thereby reweighting and calibrating the local symptom semantics in ${\hat{T}}_{s}$. The attention output is then added back to ${\hat{T}}_{s}$ in a residual manner to obtain the intermediate textual feature *T_r_*. In Equation 23, LN(·) denotes layer normalization, FFN(·) denotes the feed-forward network, and *T_out_* represents the final enhanced textual feature.In Equation 24, *X* serves as the query, while the visual prototypes *P* serve as the key and value, enabling potential symptom prototypes to be fed back into local patch representations. $\hat{X}$ denotes the enhanced patch features, γ_res_ is a learnable gating parameter in the patch feature feedback branch, and σ(γ_res_) denotes the visual residual scaling weight constrained by the Sigmoid function, which controls the strength of prototype feedback. Finally, the enhanced text feature *T_out_* and the enhanced patch features $\hat{X}$ are jointly used for subsequent diseased score calculation.

To prevent a small number of visual prototypes from dominating the responses over time, a prototype usage regularization term is further introduced. Unlike Equation 17, which aggregates high-response patches along the spatial dimension, the prototype usage regularization term measures the usage of different prototypes for each patch along the prototype dimension. The computation is formulated in Equations 25–27 as follows:

(25) $$U_{n,\;k}=\frac{\exp(R_{n,\;k}/\tau_{g})}{\sum_{j=1}^{K}\exp(R_{n,\;j}/\tau_{g})}$$

(26) $${\bar{u}}_{k}=\frac{1}{N}\sum_{n=1}^{N}U_{n,\;k}$$

(27) $$L_{proto}=\sum_{k=1}^{K}{\bar{u}}_{k}log\frac{{\bar{u}}_{k}}{1/K}$$

Here, τ_g_ denotes the global temperature coefficient, *U_n, k_* denotes the normalized usage probability of the *n*-th patch for the *k*-th visual prototype, ${\bar{u}}_{k}$ denotes the average usage ratio of the *k*-th visual prototype across all patches, and $L_{proto}$ denotes the prototype usage regularization term. This regularization term constrains the prototype usage distribution to be close to a uniform distribution, thereby alleviating prototype collapse caused by the overuse of a few prototypes and maintaining diversity among different visual prototypes. Overall, ACPAM extracts potential symptom prototypes through spatially sharpened aggregation and uses them for text feature enhancement and patch feature feedback, enabling the model to better exploit local disease cues during image-level disease discrimination and reduce the interference of complex backgrounds on disease scores.

### 3.5 Disease score calculation and source-domain optimization

After the preceding modules, the model obtains the enhanced patch features $\hat{X}$ and the enhanced text features *T_out_*. Here, $\hat{X}={\left\{{\hat{x}}_{n}\right\}}_{n=1}^{N}$, where ${\hat{x}}_{n}$ denotes the enhanced visual feature of the *n*-th patch, and *N* denotes the number of patches. The enhanced text feature *T_out_* contains healthy and diseased semantics, denoted as *t*_0_ and *t*_1_, respectively. This section mainly consists of two parts: the disease score calculation path in the inference stage, which derives image-level disease scores from local patch responses, and the source-domain optimization path in the training stage, which optimizes the learnable modules using source-domain supervision and regularization terms.

Specifically, the model first computes the similarity between each patch and the healthy/diseased text semantics, and then obtains the disease-channel probability through Softmax. Considering that field diseases usually appear only in local image regions, this paper does not directly average the responses of all patches, but instead selects the *K_top_* patches with the highest disease responses for multiple-instance aggregation to obtain the image-level disease score. Meanwhile, during source-domain training, the model is jointly optimized using the image-level classification loss, source-domain pixel-level auxiliary loss, the KL divergence constraint of UPFM, and the prototype usage regularization of ACPAM. The patch-level disease probability, image-level disease score, and total loss are formulated in Equations 28–30, respectively:

(28) $$a_{n}=\text{Softmax}{\left(\frac{\left[sim\left({\hat{x}}_{n},\;t_{0}\right),\;sim\left({\hat{x}}_{n},\;t_{1}\right)\right]}{\tau }\right)}_{1}$$

(29) $$S_{img}=\frac{1}{K_{top}}\sum_{a_{n}\in TopK\left({\left\{a_{n}\right\}}_{n=1}^{N}\right)}a_{n}$$

(30) $$L=L_{cls}^{s}+\lambda_{pix}L_{pix}^{s}+\lambda_{KL}L_{KL}+\lambda_{proto}L_{proto}$$

Here, *sim*(·, ·) denotes the similarity calculation, *τ* denotes the similarity temperature coefficient, and subscript 1 indicates the disease channel. *a_n_* denotes the disease response of the *n*-th patch, and a larger value indicates that the patch is more likely to correspond to an abnormal or suspected diseased region. *K_top_* denotes the number of selected high-response patches, TopK(·) selects the *K_top_* highest responses from all patch-level disease responses, and *S_img_* denotes the image-level disease score. This score is used for the final image-level healthy/diseased discrimination.

Equation 30 defines the total loss during source-domain training. Here, $L_{cls}^{\;s}$ denotes the source-domain image-level classification loss for optimizing the normal/anomalous binary classification result, and $L_{pix}^{s}$ denotes the source-domain pixel-level auxiliary loss for constraining local anomaly responses. ${ℒ}_{\text{KL}}$ denotes the KL divergence constraint in UPFM, which limits the deviation of probabilistic prompt distributions, while ${ℒ}_{\text{proto}}$ denotes the prototype usage regularization term in ACPAM, which alleviates over-concentration of visual prototypes. λ_pix_, λ_KL_, and λ_proto_ are the corresponding loss weights.During training, the CLIP visual encoder and text encoder remain frozen, and only MGSAM, UPFM (including its learnable prompt-distribution parameters), and ACPAM are updated.

The disease score calculation path and the source-domain optimization path are functionally connected. The disease score calculation path determines how the model derives image-level predictions from local patch responses, enabling the model to focus on a small number of high-response suspected diseased regions during inference and reduce the dilution of disease scores caused by background patches.The source-domain optimization path constrains local anomaly responses using source-domain image-level labels and pixel-level masks, and stabilizes the learning of UPFM and ACPAM through the KL constraint and prototype regularization.After training, target agricultural images are only processed through forward inference to obtain *S_img_*, without using target-domain pixel-level lesion masks, thereby maintaining the target-domain-free experimental setting.

## 4 Results

### 4.1 Experimental protocol

To evaluate the zero-shot disease discrimination capability of MSPRP on target agricultural datasets, a source-domain training and target-domain testing protocol is adopted. Model optimization, hyperparameter selection, and checkpoint selection are performed exclusively using source-domain data. Because the official training partition of MVTec AD contains only normal images, whereas the source-domain supervision used in this study requires both normal and anomalous samples, an internal source-domain training/validation split is constructed from the official MVTec AD test partition. Specifically, the official test partition contains 1, 725 images, including 467 normal images and 1, 258 anomalous images. Using a fixed random seed of 111, these images are stratified by object category and anomaly status and divided into an 80% source-training set and a 20% source-validation set. The resulting source-training set contains 1, 380 images (374 normal and 1, 006 anomalous), while the source-validation set contains 345 images (93 normal and 252 anomalous), with no overlap between the two subsets.

During source-domain training, normal/anomalous image-level labels are used for image-level supervision. The official pixel-level anomaly masks provided for anomalous MVTec AD images are used for the source-domain pixel-level auxiliary loss, while normal images are assigned all-zero masks. The source-validation set is used only for source-side model selection and hyperparameter selection. After source-domain training, the selected model is fixed and directly evaluated on the four agricultural target datasets. No target-domain images or labels are used for model training, fine-tuning, hyperparameter selection, or checkpoint selection.

### 4.2 Datasets

MVTec AD, which contains images and pixel-level annotations, is used as the source-domain training dataset (Bergmann et al., 2019).The use of an industrial anomaly dataset as the source domain is motivated by the structural similarity between industrial defects and visible plant disease symptoms at the anomaly-detection level rather than by semantic similarity between the two domains. In both cases, abnormal regions often appear as localized deviations from an otherwise regular visual pattern, such as changes in texture, color, surface structure, or boundary appearance. MVTec AD provides diverse normal/anomalous image pairs together with pixel-level anomaly masks across multiple object and texture categories, allowing the model to learn generic local anomaly-response patterns without relying on agricultural target-domain images. These source-domain anomaly cues are subsequently transferred through the frozen CLIP representation space, while the proposed modules adapt the learned responses to multi-scale symptoms, prompt uncertainty, and background interference in agricultural images. Therefore, MVTec AD is used as a generic source of anomaly supervision rather than as a semantic surrogate for plant diseases. MVTec AD contains 15 industrial object and texture categories. Its official training partition contains 3, 629 normal images, whereas the official test partition contains 1, 725 images, comprising 467 normal and 1, 258 anomalous images. Pixel-level anomaly masks are provided for the 1, 258 anomalous test images. In this study, MVTec AD serves exclusively as the source domain rather than as an evaluation benchmark. Because the official training partition contains no anomalous samples, the official test partition is repurposed as the labeled source-domain pool and is further divided into the internal source-training and source-validation subsets described in Section 4.1. The official anomaly masks are used only for source-domain pixel-level supervision.Representative normal and anomalous source-domain MVTec AD images together with their corresponding anomaly masks are shown in **Figure 5**.

**Figure 5:**
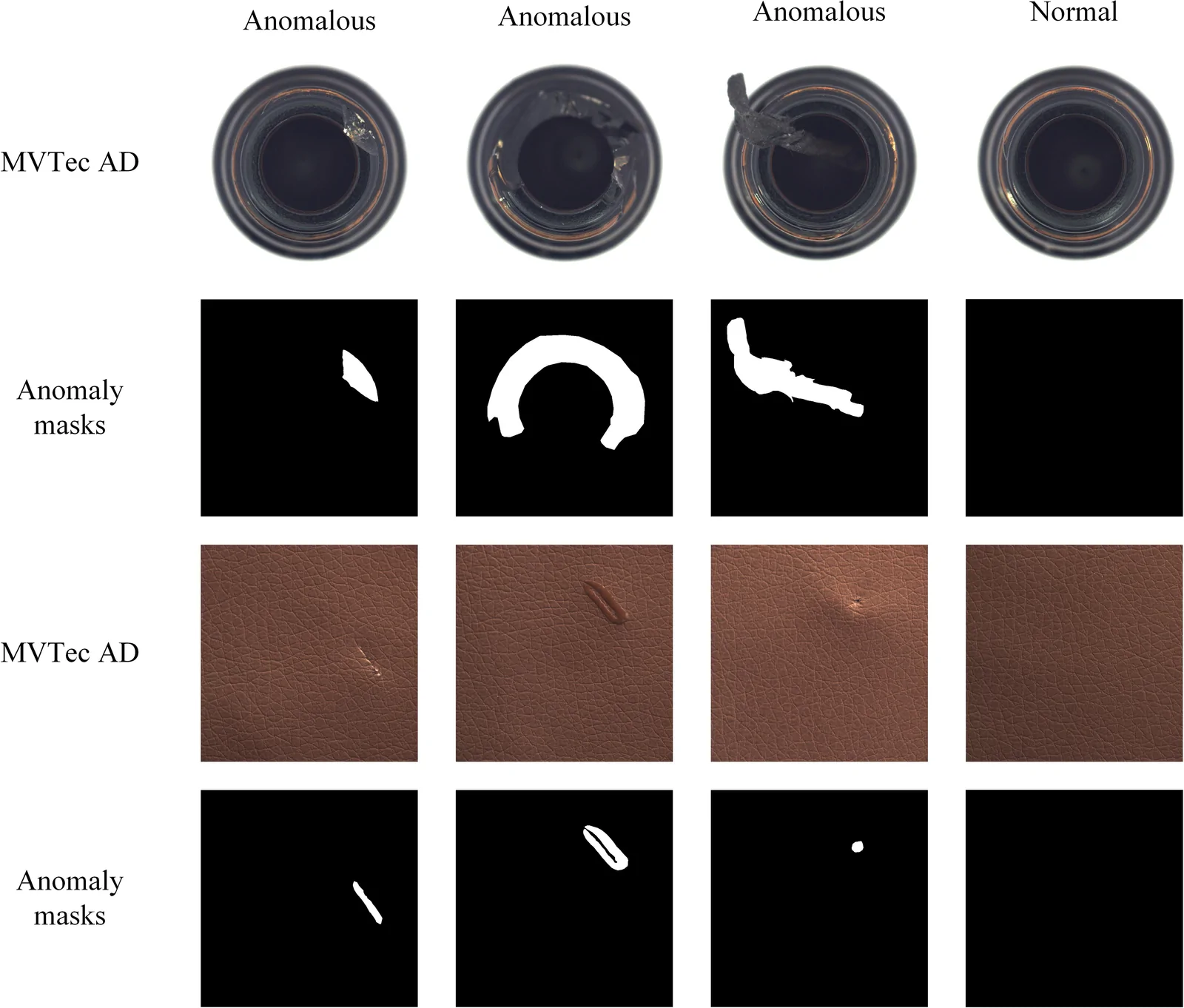
Representative normal and anomalous source-domain images from MVTec AD and their corresponding official pixel-level anomaly masks. For visualization consistency, normal samples are paired with all-zero masks.

The target domain contains four agricultural datasets, including three public datasets, namely OLID I (Orka et al., 2023), SLIF-Brinjal (George et al., 2026), and HibiscusTea (Billah et al., 2026), as well as a field *Polygonatum odoratum* disease dataset constructed in this study. The field dataset was collected during seven non-consecutive field visits from April to June under real planting conditions. Image acquisition covered both sunny and overcast weather conditions and relied primarily on natural illumination, without additional artificial lighting or controlled backgrounds. Images were captured handheld by multiple collectors using smartphones from domestic brands, including Huawei and Xiaomi. To preserve the variability of practical field acquisition, no strictly fixed imaging distance or viewing angle was imposed; instead, images were captured at practical handheld distances that allowed the target leaves or plants to be clearly observed. The resulting dataset contains 90 healthy images and 95 diseased images and retains natural variations in illumination, background, imaging distance, and viewpoint that commonly occur in real field scenarios.Dataset statistics are summarized in **Table 1**, and representative images are shown in **Figure 6**. The datasets differ in leaf shape, background, image quality, and lesion appearance.

**Table 1:** Dataset statistics for source-domain training/validation and target-domain evaluation.

Dataset	Domain	Split	Classes	Normal/ healthy	Anomalous/ diseased	Usage
MVTec AD	Source	Source-train	15 Industrial	374	1006	Source-domain optimization
MVTec AD	Source	Source-val	15 Industrial	93	252	Source-only model selection
OLID I	Target	Test	8 Vegetables	786	3963	Target-domain evaluation
SLIF-Brinjal	Target	Test	1 Brinjal	1090	7897	Target-domain evaluation
HibiscusTea	Target	Test	2 Hibiscus/Tea	521	890	Target-domain evaluation
Polygonatum	Target	Test	1 Polygonatum	90	95	Target-domain evaluation

**Figure 6:**
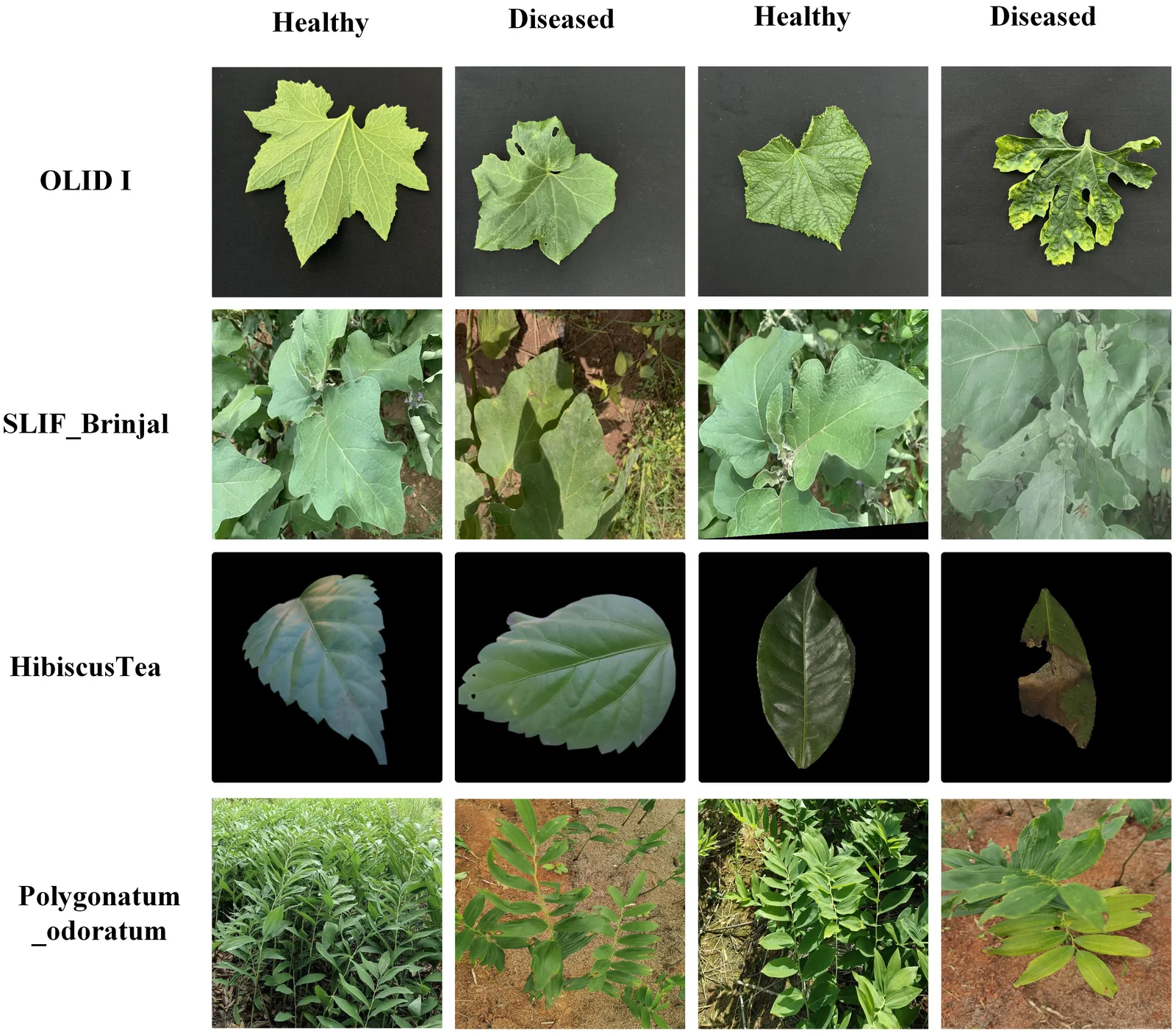
Image examples from the four agricultural datasets.

Sample independence and duplicate-image audit. For the self-constructed Polygonatum odoratum dataset, each image was acquired from a different physical plant, and the same plant was not repeatedly photographed from multiple viewpoints. For the three public target datasets, plant- or specimen-level identifiers are not available in the public metadata and documentation; therefore, plant-level independence cannot be retrospectively verified, and possible correlations caused by repeated or multi-view imaging cannot be completely excluded.

To further examine potential image-level redundancy, an additional duplicate-image audit was conducted using exact file matching and perceptual similarity screening. One exact cross-label duplicate pair was identified in OLID I and one in HibiscusTea, whereas no corresponding exact cross-label duplicate was identified in SLIF-Brinjal or the Polygonatum odoratum dataset. As a robustness check, both images belonging to each identified cross-label duplicate pair were excluded, resulting in cleaned evaluation sets containing 4, 747 images for OLID I and 1, 409 images for HibiscusTea. The fixed model was then reevaluated without retraining or parameter adjustment, and only marginal changes in the evaluation metrics were observed. These results indicate that the isolated duplicate pairs do not materially affect the overall conclusions.

### 4.3 Evaluation metrics

Image-level area under the receiver operating characteristic curve (AUROC) and average precision (AP) are used as the main evaluation metrics in this study.AUROC is the area under the ROC curve and is used to measure the model’s ability to distinguish healthy samples from diseased samples under different decision thresholds. This metric reflects the degree of separation of disease scores between the two classes of samples. A higher AUROC indicates that the model is more capable of assigning higher disease scores to diseased samples and separating them from healthy samples. AP is the area under the Precision-Recall curve and is used to evaluate the model’s ranking performance for diseased samples. A higher AP indicates that diseased samples are ranked higher in the prediction order, suggesting better recognition performance for diseased samples. During calculation, the disease score and image-level ground-truth label of each test image are used for statistical evaluation, and all metric results are reported as percentages.

In addition to the threshold-independent area under the receiver operating characteristic curve (AUROC) and average precision (AP), sensitivity, specificity, balanced accuracy, and F1 score are reported as supplementary threshold-dependent metrics. Sensitivity measures the proportion of diseased samples correctly identified, whereas specificity measures the proportion of healthy samples correctly identified. Balanced accuracy is calculated as the average of sensitivity and specificity, thereby reducing the influence of class imbalance. The F1 score provides a harmonic mean of precision and recall for the diseased class.

To preserve the target-domain-free evaluation setting, the classification threshold is determined exclusively using the internal source-domain validation set. The threshold is selected by maximizing balanced accuracy, with the F1 score used as a tie-breaker when necessary. Once determined, the same threshold is fixed and applied unchanged to all four target agricultural datasets. No target-domain images or labels are used for threshold selection, adjustment, or recalibration.

### 4.4 Experimental settings

The experiments are implemented using PyTorch (Paszke et al., 2019) and run on an NVIDIA GeForce RTX 3090 GPU. The model backbone adopts the pretrained CLIP ViT-L/14 dual-encoder architecture (Radford et al., 2021), consisting of a visual encoder and a text encoder. During training, the CLIP visual encoder and text encoder remain frozen, and only MGSAM, UPFM (including its learnable prompt-distribution parameters), and ACPAM are updated.The input image size is set to 518×518. The visual branch extracts patch features from the 6th, 12th, 18th, and 24th layers for multi-granularity symptom modeling. The Adam optimizer (Kingma and Ba, 2015) is used with an initial learning rate of 0.001, a batch size of 16.All models are trained for 15 epochs, and a warmup strategy is adopted during the early training stage to stabilize the optimization process. Model selection is performed exclusively using the internal source-domain validation set. For the full MSPRP model, the checkpoint at epoch 12 is selected according to the source-domain validation AUROC and is then fixed for evaluation on all target agricultural datasets. No target-domain images or labels are used for checkpoint selection.For the supplementary threshold-dependent evaluation, the decision threshold is likewise selected exclusively on the internal source-domain validation set and is then fixed for all target-domain evaluations.The loss function consists of the source-domain image-level classification loss, the source-domain pixel-level auxiliary loss, the Kullback–Leibler (KL) divergence constraint of UPFM, and the prototype usage regularization of ACPAM.The KL loss weight is set to 0.0001 based on the sensitivity analysis conducted on the internal source-domain validation set, while the prototype regularization weight is set to 0.01. The number of visual prototypes in ACPAM is set to 2 based on the sensitivity analysis conducted on the internal source-domain validation set.During inference, Top-*K* multi-instance scoring is adopted, with *K_top_* set to 10.For the repeated leave-one-module-out ablation analysis reported in **Table 2**, each configuration is independently trained using three random seeds (111, 222, and 333), and epoch 12 is consistently used for evaluation to maintain the same checkpoint protocol. The repeated results are reported as mean ± standard deviation. The hyperparameter and backbone sensitivity analyses reported in **Tables 3**–**5** are conducted separately as independent single-run experiments under the same fixed source-domain train/validation split.

**Table 2:** Stability analysis of the main leave-one-module-out ablations over three independent runs.

Configuration	Source-val AUROC	Source-val AP	Target mean AUROC	Target mean AP
Full MSPRP	99.35 ± 0.14	99.47 ± 0.12	79.60 ± 1.10	89.35 ± 1.13
w/o MGSAM	97.77 ± 0.76	98.72 ± 0.51	76.53 ± 0.30	87.44 ± 1.22
w/o UPFM	99.10 ± 0.29	99.31 ± 0.12	76.19 ± 0.03	88.03 ± 1.67
w/o ACPAM	99.17 ± 0.14	99.36 ± 0.16	75.56 ± 1.03	87.54 ± 1.80

Results are reported as mean ± standard deviation (%). Target-domain results represent the macro average over OLID I, SLIF-Brinjal, HibiscusTea, and Polygonatum odoratum.

**Table 3:** Single-run sensitivity analysis of the number of visual prototypes in ACPAM on the internal source-domain validation set.

Number of prototypes	AUROC (%)	AP (%)
1	98.83	99.07
2	99.48	99.78
4	99.32	99.54
8	99.28	99.50

**Table 4:** Single-run sensitivity analysis of the KL-divergence loss weight in UPFM on the internal source-domain validation set.

KL loss weight λ_KL_	AUROC (%)	AP (%)
1×10^−5^	99.01	99.32
1×10^−4^	99.48	99.78
1×10^−3^	99.32	99.31

**Table 5:** Single-run backbone sensitivity analysis under the source-only selection protocol.

CLIP backbone	Selected layers	Feature dim.	Source-val AUROC	Source-val AP	Target mean AUROC	Target mean AP
ViT-B/32	3/6/9/12	512	96.79	98.69	71.33	82.64
ViT-B/16	3/6/9/12	512	96.99	98.29	72.22	82.51
ViT-L/14	6/12/18/24	768	99.48	99.78	78.70	90.35

### 4.5 Comparative experiments

To evaluate the image-level healthy/diseased discrimination performance of MSPRP on target agricultural datasets, four categories of methods are selected for comparison.The first category consists of traditional industrial anomaly detection methods, including PaDiM (Defard et al., 2021) and PatchCore (Roth et al., 2022); the second category is the general vision-language model baseline CLIP (Radford et al., 2021); the third category includes training-free or zero-shot anomaly detection methods, including WinCLIP (Jeong et al., 2023), SAA (Cao et al., 2023), and MuSc (Li et al., 2024); and the fourth category includes prompt learning or anomaly semantic adaptation methods, including AnomalyCLIP (Zhou et al., 2024), CLIP-AD (Chen et al., 2025), and TPS (Ma et al., 2025).

It should be noted that CLIP, WinCLIP, SAA, MuSc, AnomalyCLIP, CLIP-AD, TPS, and the proposed method do not use any target agricultural training images for training. Specifically, training-free methods do not perform additional parameter training, while source-trained target-domain-free methods are trained on source-domain data but do not use target agricultural images for training or fine-tuning. PaDiM and PatchCore adopt a 5-shot target-normal setting, in which five target-domain normal images from each target category are used to establish a normal feature distribution or memory bank.As shown in **Table 6**, bold values indicate the results of the proposed MSPRP, and underlined values indicate the best results among the comparison methods that do not use target agricultural training images. Methods marked with † use 5-shot target-domain normal samples for modeling and are included only as reference results under the condition of limited target-domain healthy samples, without being included in the ranking of target-domain-free methods.

**Table 6:** Image-level disease detection performance on four target plant disease datasets. Results are reported as AUROC/AP (%).

Method	Training setting	OLID I	SLIF-Brinjal	Hibiscustea	Polygonatumodoratum	Mean
PaDiM†	5-shot target-normal	56.7/87.8	45.4/87.4	46.8/69.6	70.7/74.0	54.9/79.7
PatchCore†	5-shot target-normal	73.0/95.5	75.8/97.3	68.3/90.0	82.6/92.8	74.9/93.9
CLIP	Training-free zero-shot	63.8/90.6	83.6/97.1	67.3/79.3	64.5/73.1	69.8/85.0
WinCLIP	Training-free zero-shot	67.2/91.6	74.4/95.7	64.3/78.9	32.2/39.4	59.5/76.4
SAA	Training-free zero-shot	61.5/87.9	53.2/90.0	59.1/79.2	52.0/59.0	56.5/79.0
MuSc	Training-free zero-shot	62.6/89.4	83.9/96.8	62.5/79.3	79.5/80.0	72.1/86.4
TPS	Source-trained target-domain-free zero-shot	74.1/93.5	81.1/97.2	72.8/83.1	68.3/76.4	74.1/87.6
AnomalyCLIP	Source-trained target-domain-free zero-shot	73.9/93.9	80.5/97.0	70.6/82.2	66.5/70.8	72.9/86.0
CLIP-AD	Source-trained target-domain-free zero-shot	67.5/91.2	74.5/95.7	75.8/84.1	33.9/40.5	62.9/77.9
*MSPRP*	Source-trained target-domain-free zero-shot	75.2/93.8	82.6/97.4	77.0/85.5	80.0/84.7	78.7/90.4

† indicates methods using 5-shot target-domain normal samples for modeling; these methods are included only as reference results and are not included in the ranking of target-domain-free methods.

For the comparative experiments, each baseline is implemented following its original paper or publicly released configuration rather than forcibly replacing its backbone and preprocessing pipeline with those of MSPRP. Accordingly, the backbone architecture, input resolution, and preprocessing procedure are method-dependent. The original resizing/cropping and normalization procedures specified by each implementation are retained. For representative configurations, AnomalyCLIP and TPS use a ViT-L/14-based CLIP backbone with 518 × 518 inputs, whereas CLIP-AD uses the ViT-B-16-plus-240 backbone with 240 × 240 inputs. MuSc is evaluated using its ViT-L-14–336 configuration with an input size of 518 × 518, while PaDiM and PatchCore retain their original CNN-based feature extraction and preprocessing pipelines. The remaining baselines likewise follow the input transformation and normalization settings of their public implementations. All methods are evaluated under the same target-dataset evaluation protocol using the same image-level ground-truth labels and the same AUROC and AP definitions. No additional agricultural-target-specific preprocessing, target-domain parameter tuning, or fine-tuning beyond the original implementation is introduced. Accordingly, **Table 6** should be interpreted as a comparison among representative implementations of the different methods rather than a strictly backbone-controlled architectural comparison. The effect of the CLIP backbone itself is separately examined under a controlled protocol in Section 4.8.

**Table 6** shows that, among methods that do not use target agricultural training images, MSPRP achieves the highest average AUROC and AP, with scores of 78.7% and 90.4%, respectively.

Compared with TPS and SAA, the proposed method improves the average AUROC and AP by 4.6 and 2.8 percentage points, and by 22.2 and 11.4 percentage points, respectively.These results indicate that MSPRP can improve image-level disease discrimination when transferring the model from the industrial source domain to agricultural target domains.

From the perspective of different method categories, the general CLIP baseline shows a certain disease discrimination capability on some datasets, but its performance fluctuates across different datasets. Training-free zero-shot methods such as WinCLIP, SAA, and MuSc do not require additional parameter training; among them, MuSc achieves a high AUROC on SLIF-Brinjal but shows relatively limited performance on OLID I and HibiscusTea. Prompt-based zero-shot anomaly detection methods such as AnomalyCLIP, CLIP-AD, and TPS enhance disease-aware capability through disease semantics or prompt adaptation, resulting in relatively stable overall performance. In contrast, MSPRP maintains relatively high AUROC or AP values across the four target agricultural datasets, indicating its applicability to different agricultural image scenarios.

The performance of MSPRP nevertheless varies across the four target agricultural datasets. Its AUROC ranges from 75.2% to 82.6%, reaching 82.6% on SLIF-Brinjal, 80.0% on Polygonatum odoratum, 77.0% on HibiscusTea, and 75.2% on OLID I. Such differences may be related to variations in crop species, symptom appearance, background complexity, imaging conditions, and intra-class visual diversity across the datasets. Since the model is trained only on industrial anomaly data, the degree of distribution shift between the source domain and each agricultural target domain is not identical, which can lead to different transfer performance.

The variation in AP is more pronounced than that in AUROC. Diseased samples account for approximately 87.9% of SLIF-Brinjal and 83.5% of OLID I, compared with approximately 63.1% of HibiscusTea and 51.4% of Polygonatum odoratum. Because AP is sensitive to the prevalence of the positive class, the relatively high proportion of diseased samples in SLIF-Brinjal and OLID I can contribute to higher AP values. Therefore, the lower AP of Polygonatum odoratum (84.7%), despite its relatively high AUROC of 80.0%, should not be interpreted directly as substantially weaker disease discrimination. For this reason, both AUROC and AP are reported and interpreted together when comparing performance across target datasets.

The results of PaDiM and PatchCore reflect the performance of traditional anomaly detection methods under the condition of a small number of target-domain healthy samples. PatchCore achieves high AP values on Polygonatum odoratum, OLID I, and HibiscusTea, indicating that target-domain healthy samples can provide direct support for normal feature modeling. However, such methods require target agricultural healthy images to establish feature distributions or memory banks, which differs from the setting of this study where no target agricultural training images are used. In comparison, MSPRP obtains image-level disease scores only through forward inference and does not rely on target-domain training samples or target-domain pixel-level lesion annotations, making it more consistent with practical field application requirements.

As shown in **Table 7**, using the fixed threshold selected exclusively from the source-domain validation set, MSPRP achieves a macro-averaged sensitivity of 77.10%, specificity of 67.31%, balanced accuracy of 72.20%, and F1 score of 82.52% across the four target agricultural datasets. The results show that the model maintains a reasonable trade-off between disease sensitivity and healthy-sample specificity without target-domain threshold calibration, although the operating-point performance varies across datasets. SLIF-Brinjal achieves the highest balanced accuracy of 78.29%, whereas P. odoratum exhibits the most balanced sensitivity and specificity, reaching 76.04% and 74.23%, respectively. These threshold-dependent metrics provide complementary information about model behavior at a fixed operating point, while AUROC and AP remain the primary metrics for evaluating threshold-independent discrimination and ranking performance.

**Table 7:** Threshold-dependent classification performance on the four target agricultural datasets using a source-domain-selected fixed threshold.

Dataset	Sensitivity (%)	Specificity (%)	Balanced accuracy (%)	F1 (%)
OLID I	76.20	60.01	68.11	87.36
SLIF-Brinjal	84.93	71.64	78.29	92.02
HibiscusTea	71.21	63.36	67.29	75.89
*P. odoratum*	76.04	74.23	75.14	74.81
Macro mean	77.10	67.31	72.20	82.52

The classification threshold is selected exclusively on the internal source-domain validation set and is then fixed for all target agricultural datasets. No target-domain images or labels are used for threshold selection or adjustment.

### 4.6 Ablation study

To analyze the effects of ACPAM, MGSAM, and UPFM on model performance, ablation experiments were conducted on four datasets: OLID I, SLIF-Brinjal, HibiscusTea, and *Polygonatum odoratum*. Under the same baseline setting, different module combinations were incrementally introduced for evaluation, and the results are reported in **Table 8**.

**Table 8:** Quantitative results of the single-run module combination ablation study for MSPRP.

Proposed modules			OLID I	SLIF-Brinjal	HibiscusTea	Polygonatum odoratum
ACPAM	MGSAM	UPFM				
***baseline***			74.1/93.5	81.1/97.2	72.8/83.1	68.3/76.4
**√**			74.5/93.7	81.5/97.2	75.5/85.1	71.3/76.6
	**√**		74.5/93.6	81.3/97.2	75.8/85.3	71.1/76.1
		**√**	74.4/93.6	81.5/97.2	74.1/84.2	71.2/76.5
**√**	**√**		74.9/**93.8**	81.7/97.3	76.6/85.3	71.6/76.4
**√**		**√**	74.8/**93.8**	82.2/97.3	76.1/85.2	72.8/78.7
	**√**	**√**	74.9/93.7	82.3/97.3	76.5/**86.0**	72.0/77.2
**√**	**√**	**√**	**75.2/93.8**	**82.6/97.4**	**77.0/**85.5	**80.0/84.7**

Results are reported as AUROC/AP (%), and the best result for each metric is highlighted in bold.

Taking the baseline without any additional modules as the reference, adding MGSAM alone improves the AUROC on OLID I, SLIF-Brinjal, HibiscusTea, and *Polygonatum odoratum* from 74.1%, 81.1%, 72.8%, and 68.3% to 74.5%, 81.3%, 75.8%, and 71.1%, respectively. Among these datasets, the improvements on HibiscusTea and *Polygonatum odoratum* are more pronounced, with increases of 3.0 and 2.8 percentage points, respectively. The AP metric is also improved on OLID I and HibiscusTea. These results indicate that adding MGSAM improves AUROC on multiple datasets, suggesting that this module helps enhance the model’s ability to distinguish healthy samples from diseased samples.

After adding UPFM alone, the AUROC values on the four datasets reach 74.4%, 81.5%, 74.1%, and 71.2%, respectively, all of which are higher than those of the baseline. Specifically, the AUROC increases by 2.9 percentage points on *Polygonatum odoratum*, 1.3 percentage points on HibiscusTea, and 0.4 percentage points on SLIF-Brinjal. The AP metric also shows slight improvements on OLID I, HibiscusTea, and *Polygonatum odoratum*. These results indicate that adding UPFM improves disease discrimination on multiple target agricultural datasets, suggesting that probabilistic prompt modeling has a positive effect on improving image-level disease detection performance under cross-domain testing.

After adding ACPAM alone, the AUROC values on the four datasets increase to 74.5%, 81.5%, 75.5%, and 71.3%, respectively. Compared with the baseline, the AUROC on HibiscusTea increases by 2.7 percentage points and the AP increases from 83.1% to 85.1%; on *Polygonatum odoratum*, the AUROC increases by 3.0 percentage points and the AP increases from 76.4% to 76.6%; and on OLID I, the AUROC and AP increase to 74.5% and 93.7%, respectively. This indicates that adding ACPAM improves AUROC or AP on multiple datasets, suggesting that cross-modal prototype aggregation has a positive effect on image-level disease discrimination and disease sample ranking.

Overall, the single-run ablation results in **Table 8** show that MGSAM, UPFM, and ACPAM contribute to image-level disease discrimination to different degrees. To further assess whether these improvements are robust to random initialization and stochastic optimization, we additionally repeated the main leave-one-module-out experiments using three independent random seeds (111, 222, and 333). The results are reported as mean ± standard deviation in **Table 2**.

As shown in **Table 2**, the full MSPRP achieves a target-domain mean AUROC of 79.60 ± 1.10% and a mean AP of 89.35 ± 1.13%, achieving higher mean target-domain performance than all three leave-one-module-out configurations. Removing MGSAM reduces the target-domain mean AUROC and AP to 76.53 ± 0.30% and 87.44 ± 1.22%, respectively, corresponding to decreases of 3.07 and 1.91 percentage points. Removing UPFM yields a mean AUROC of 76.19 ± 0.03% and a mean AP of 88.03 ± 1.67%, representing decreases of 3.41 and 1.32 percentage points, respectively. Removing ACPAM reduces the mean AUROC and AP to 75.56 ± 1.03% and 87.54 ± 1.80%, respectively, corresponding to decreases of 4.04 and 1.81 percentage points. The full model also achieves the highest mean source-validation AUROC and AP among the four configurations. Overall, removing any of the three modules reduces mean target-domain performance. The largest AUROC reduction is observed when ACPAM is removed, whereas the largest AP reduction occurs when MGSAM is removed, indicating that the three modules provide complementary contributions across different evaluation metrics.

### 4.7 Model complexity and efficiency analysis

As shown in **Table 9**, compared with the baseline, MSPRP introduces an additional 10.65 M parameters, corresponding to an increase of approximately 2.49%. The additional parameters mainly come from MGSAM, UPFM, and ACPAM, with MGSAM contributing 8.12 M parameters, UPFM contributing 0.02 M parameters, and ACPAM contributing 2.51 M parameters. Under the same software and hardware conditions, the single-image inference latency of MSPRP is 108.19 ms, which is close to that of the baseline. These results indicate that the additional computational overhead introduced by MGSAM, UPFM, and ACPAM is relatively limited compared with the baseline. However, the current MSPRP implementation still relies on the large-scale CLIP ViT-L/14 backbone, resulting in a total model size of 438.59 M parameters. Therefore, the limited overhead of the proposed modules should not be interpreted as evidence that the complete model is already suitable for resource-constrained mobile or edge devices. In addition, the latency measurements in **Table 9** were obtained on an NVIDIA GeForce RTX 3090 GPU and mainly reflect the relative computational overhead of the proposed modules rather than actual edge-device performance.

**Table 9:** Computational efficiency comparison on an NVIDIA GeForce RTX 3090 GPU with an input image size of 518×518.FPS denotes frames per second.

Method	Backbone	Added params (M)	Total params (M)	Latency (ms)	FPS
Baseline	ViT-L/14	-	427.94	108.15	9.25
**MSPRP**	ViT-L/14	**10.65**	**438.59**	**108.19**	**9.24**
*Relative Overhead (vs. Baseline)*		+2.49%	+2.49%	+0.04%	-0.11%

Bold values indicate the results of the proposed MSPRP method.

### 4.8 Hyperparameter and Backbone Sensitivity Analysis

Additional independent single-run sensitivity analyses are conducted to examine the effects of key hyperparameters and the choice of CLIP backbone under the source-only selection protocol. The experiments reported in **Tables 3**-**5** are performed separately under the fixed source-domain train/validation split and are independent of the three-seed repeated leave-one-module-out analysis reported in **Table 2**.

To determine the number of visual prototypes in ACPAM without using target-domain information, we evaluated K∈{1, 2, 4, 8} on the internal source-domain validation set. As shown in **Table 3**, K = 2 achieves the highest validation AUROC of 99.48% and AP of 99.78%. Increasing the number of prototypes to 4 or 8 does not provide further improvement, while using a single prototype yields lower performance. Therefore, K = 2 is adopted in all experiments.

The sensitivity of UPFM to the KL-divergence loss weight λ_KL_ is further analyzed, where λ_KL_ controls the strength of the regularization imposed on the probabilistic prompt distributions. To avoid target-domain-based hyperparameter tuning, λ_KL_ is evaluated exclusively on the internal source-domain validation set using 1×10^−5^, 1×10^−4^, and 1×10^−3^. As shown in **Table 4**, all three settings achieve high validation performance, while λ_KL_ = 10^−4^ yields the highest AUROC of 99.48% and AP of 99.78%. A smaller weight provides weaker distribution regularization, whereas increasing the weight to 1×10^−3^ does not further improve validation performance. Therefore, λ_KL_ = 10^−4^ is adopted in all subsequent experiments.

The influence of the CLIP backbone is further evaluated using ViT-B/32, ViT-B/16, and ViT-L/14 under the same source-domain training and validation protocol. For the 12-block ViT-B/32 and ViT-B/16 backbones, features from the 3rd, 6th, 9th, and 12th layers are selected to approximately correspond to the relative network depths represented by the 6th, 12th, 18th, and 24th layers of the 24-block ViT-L/14. As shown in **Table 5**, ViT-L/14 achieves the highest source-validation AUROC and AP, reaching 99.48% and 99.78%, respectively, compared with 96.79%/98.69% for ViT-B/32 and 96.99%/98.29% for ViT-B/16. Therefore, ViT-L/14 is selected as the default CLIP backbone based exclusively on source-domain validation performance. After this source-only selection, target-domain performance is reported only for evaluation. ViT-L/14 achieves a target-domain mean AUROC of 78.70% and a mean AP of 90.35%, compared with 71.33%/82.64% for ViT-B/32 and 72.22%/82.51% for ViT-B/16. These results further indicate that the backbone selected using source-domain validation also provides stronger transferable representations for cross-domain plant disease discrimination under the current framework.

To further distinguish learnable probabilistic prompt modeling from deterministic prompt learning and simple random perturbation, three prompt strategies are compared under the same source-only training protocol. To isolate the effect of prompt modeling, MGSAM and ACPAM are kept unchanged, while only the prompt modeling strategy is replaced. All other training and evaluation settings are kept identical to those of the full MSPRP model. As shown in **Table 10**, UPFM achieves the highest mean performance across both the source-domain validation set and the agricultural target datasets. Specifically, its source-validation AUROC and AP reach 99.35% and 99.47%, respectively, exceeding the deterministic prompt baseline by 0.31 and 0.28 percentage points and the fixed Gaussian-noise baseline by 0.52 and 0.52 percentage points. More importantly, UPFM achieves a target-domain mean AUROC of 79.60% and a mean AP of 89.35%. Compared with deterministic prompt learning, the corresponding improvements are 6.31 and 2.83 percentage points, respectively. Compared with the fixed Gaussian-noise baseline, the improvements increase to 9.19 percentage points in AUROC and 4.78 percentage points in AP. The fixed Gaussian-noise strategy performs worse than the deterministic baseline on both target-domain metrics, suggesting that simply introducing fixed random perturbations during training does not necessarily improve cross-domain prompt transfer. In contrast, UPFM jointly learns the mean and variance of the prompt distributions and constrains them through KL regularization, allowing the stochastic variation introduced during training to be adaptively learned rather than manually fixed. Although some variation across random seeds remains, UPFM consistently achieves the highest mean performance across both source-validation and target-domain metrics. Moreover, its target-domain variability is substantially lower than that of the fixed Gaussian-noise strategy, further supporting the effectiveness of learnable probabilistic prompt modeling for cross-domain disease discrimination.

**Table 10:** Comparison of different prompt modeling strategies under the source-only protocol.

Prompt modeling	Learnable mean	Learnable variance	Stochastic training	KL regularization	Source-val AUROC	Source-val AP	Target mean AUROC	Target mean AP
Deterministic prompt	✓	–	–	–	99.04 ± 0.05	99.19 ± 0.15	73.29 ± 1.53	86.52 ± 0.86
Fixed Gaussian noise	✓	–	✓	–	98.83 ± 0.23	98.95 ± 0.44	70.41 ± 3.84	84.57 ± 2.38
UPFM	✓	✓	✓	✓	99.35 ± 0.14	99.47 ± 0.12	79.60 ± 1.10	89.35 ± 1.13

Results are reported as mean ± standard deviation over three random seeds.

### 4.9 Visualization analysis

To intuitively analyze the detection performance of the proposed method and other methods on agricultural images, multiple groups of agricultural test samples were selected for visual comparison.As shown in **Figure 7**, green labels indicate correct predictions, whereas red labels indicate incorrect predictions. All samples are from the target agricultural test sets and are not involved in model training. Compared with other methods, MSPRP yields correct predictions for several representative cases shown in **Figure 7**, while the last two rows illustrate challenging failure cases shared by all compared methods.

**Figure 7:**
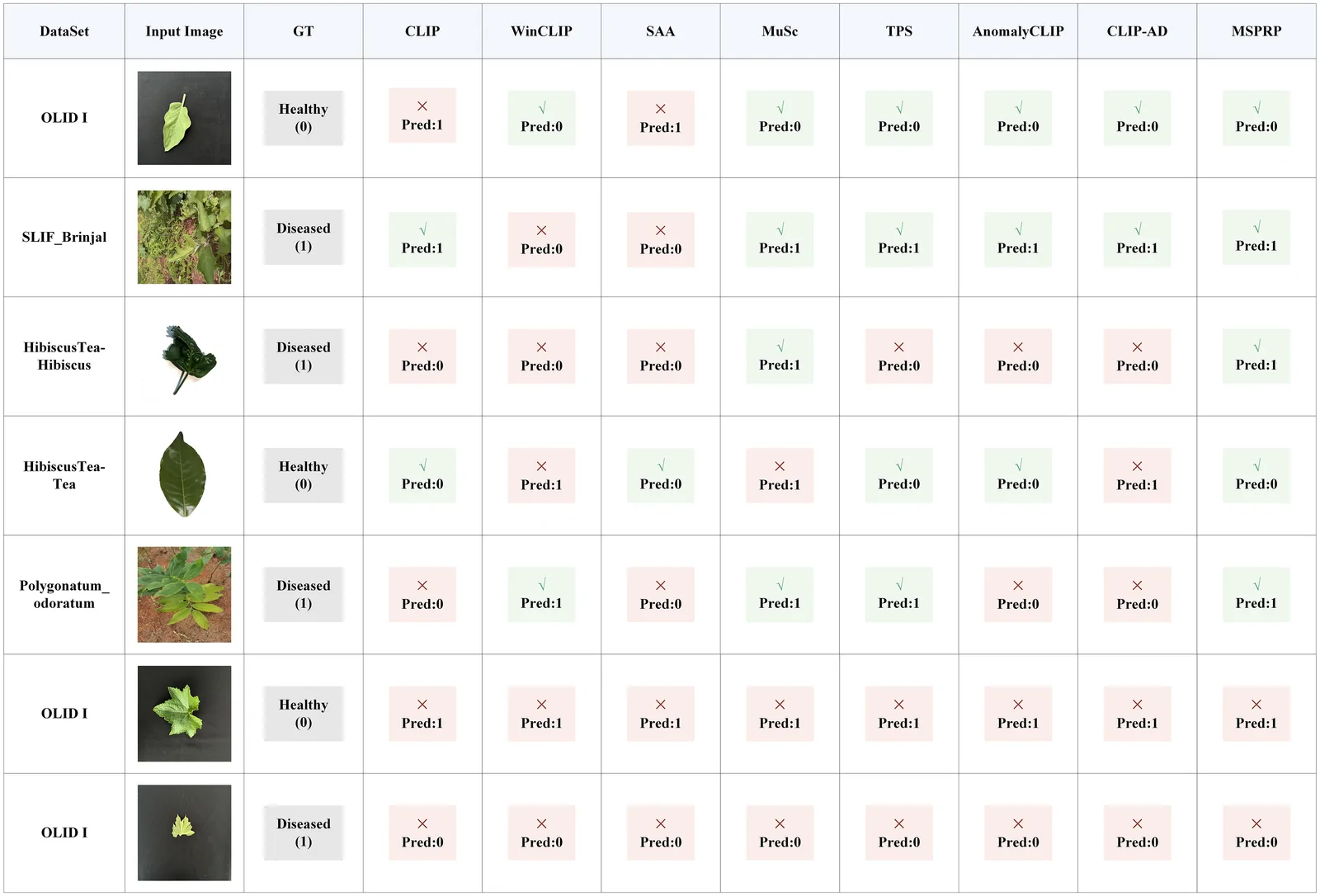
Image-level prediction results of different methods on representative and challenging test samples from the target plant disease datasets. Each row corresponds to one test image. The first three columns show the dataset, input image, and ground-truth label, respectively, while the remaining columns show the predictions of different methods. GT/Pred = 0 denotes healthy and GT/Pred = 1 denotes diseased. Green and red marks indicate correct and incorrect predictions, respectively. The last two rows show challenging cases misclassified by all compared methods.

The last two rows of **Figure 7** present two representative challenging cases that are misclassified by all compared methods. In the healthy case, prominent leaf veins, serrated boundaries, and local texture variations may produce abnormal visual responses, causing the healthy leaf to be incorrectly classified as diseased. In contrast, the diseased case exhibits relatively subtle and low-contrast visual symptoms, which may not generate sufficiently strong disease-related responses and consequently lead to a diseased sample being classified as healthy. These examples indicate that visually salient structural variations in healthy leaves and weak symptom expression in diseased leaves remain common challenges for cross-domain plant disease detection.

It should be noted that MSPRP is designed for image-level healthy/diseased discrimination rather than pixel-level lesion localization. Therefore, generic visual attention maps are not interpreted as lesion-localization evidence in this study, and systematic lesion localization evaluation is left for future work.

## 5 Discussion

The experimental results indicate that MSPRP can be used as a target-domain-free computational methodology for plant disease image analysis under field conditions. Unlike conventional plant disease recognition models that depend on target-domain training images, the proposed method is trained only on an industrial source domain and is directly evaluated on target agricultural datasets. This setting is practically relevant when a new crop, planting region, imaging device, or disease scenario is encountered and only limited target-domain annotations are available.

From a methodological perspective, the three proposed modules address different operational difficulties in field plant images. MGSAM enhances multi-layer patch features and strengthens symptom-related visual responses, which is useful when lesions appear at different scales or occupy only local leaf regions. UPFM models healthy and diseased prompt contexts as probabilistic representations, thereby reducing the rigidity of fixed textual prompts under field domain shifts. ACPAM aggregates high-response local visual prototypes and reduces the dilution of locally discriminative cues by numerous background patches in image-level disease scoring. Because the ACPAM routing stage is driven by visual patch features rather than explicit lesion annotations, visually salient background structures, such as soil, shadows, leaf boundaries, or strong illumination changes, may occasionally receive high prototype responses. The ablation results show that these components provide complementary improvements, suggesting that the final performance does not rely on a single isolated design.

The proposed method also has several limitations. First, the present study focuses on image-level healthy/diseased discrimination and does not systematically evaluate pixel-level lesion localization. Second, the self-constructed *Polygonatum odoratum* dataset is relatively small and is not yet publicly released, which limits independent external validation. Third, MSPRP does not identify specific disease categories or pathogens; instead, it provides a general healthy/diseased decision. Fourth, although the proposed modules introduce only limited additional overhead relative to the baseline, the current implementation still relies on the large-scale CLIP ViT-L/14 backbone and has not yet been optimized for resource-constrained mobile or edge devices. Fifth, the current evaluation is based on four target agricultural datasets and does not yet constitute a large-scale field deployment study. In addition, plant-level identifiers are unavailable for the three public datasets, so potential correlations caused by repeated or multi-view images of the same physical plant cannot be completely ruled out. Practical deployment may involve additional distribution shifts caused by geographic regions, seasons, crop growth stages, imaging devices, illumination conditions, and field backgrounds. Therefore, broader multi-site, multi-season, and multi-device validation is still required before large-scale practical deployment. Future work will expand the field datasets, include additional crop species and disease types, conduct broader multi-site and multi-season evaluations, investigate weakly supervised localization, and explore lightweight vision-language backbones, structured pruning, quantization, and knowledge distillation to improve both the generalization and deployability of the method in practical plant disease monitoring scenarios.

## 6 Conclusion

This paper proposes MSPRP for image-level zero-shot disease detection in the target agricultural domain under complex field scenarios. Based on frozen CLIP visual and textual encoders, the proposed method introduces a Multi-Granularity Symptom-Aware Module, an Uncertainty-Aware Prompt Flow Module, and an Adaptive Cross-Modal Prototype Aggregation Module to improve the model’s adaptability to complex field images from the perspectives of visual feature modeling, textual prompt adaptation, and cross-modal local information fusion, respectively. Unlike methods that require target-domain healthy samples for modeling or target agricultural images for training, MSPRP is trained only on industrial source-domain data, while target agricultural images are used only for inference and evaluation, thereby maintaining a target-domain-free experimental setting. Experimental results show that MSPRP achieves relatively stable image-level disease detection performance across four target agricultural datasets. Taking the field disease dataset of Polygonatum odoratum as an example, the proposed method achieves AUROC and AP values of 80.0% and 84.7%, respectively, showing competitive performance among the compared target-domain-free methods. Comparative experiments show that MSPRP achieves high average AUROC and AP under the setting where no target agricultural training images are used. The ablation experiments further demonstrate that MGSAM, UPFM, and ACPAM each bring certain performance improvements when introduced individually, and their combined use further improves the overall results, indicating that the three modules positively contribute to image-level disease detection. Overall, MSPRP can perform healthy/diseased discrimination in complex field images without using target agricultural training images or target-domain pixel-level lesion annotations, providing a feasible solution for low-annotation image-level agricultural disease screening. The current study mainly focuses on image-level disease detection and has not yet systematically evaluated pixel-level lesion localization.Future work will expand the field datasets, include additional crop species and disease types, conduct broader multi-site and multi-season evaluations, investigate weakly supervised localization, and explore lightweight vision-language backbones, structured pruning, quantization, and knowledge distillation to improve both the generalization and deployability of the method in practical plant disease monitoring scenarios.

## Data Availability

The raw data supporting the conclusions of this article will be made available by the authors, without undue reservation.
